# Complement-Mediated Neutralisation Identified in Ebola Virus Disease Survivor Plasma: Implications for Protection and Pathogenesis

**DOI:** 10.3389/fimmu.2022.857481

**Published:** 2022-04-12

**Authors:** Jack Mellors, Tom Tipton, Sarah Katharina Fehling, Joseph Akoi Bore, Fara Raymond Koundouno, Yper Hall, Jacob Hudson, Frances Alexander, Stephanie Longet, Stephen Taylor, Andrew Gorringe, N’Faly Magassouba, Mandy Kader Konde, Julian Hiscox, Thomas Strecker, Miles Carroll

**Affiliations:** ^1^ Department of Research and Evaluation, United Kingdom (UK) Health Security Agency, Salisbury, United Kingdom; ^2^ Department of Infection Biology, Institute of Infection and Global Health, University of Liverpool, Liverpool, United Kingdom; ^3^ Wellcome Centre for Human Genetics and the Pandemic Sciences Institute, Nuffield Department of Medicine, University of Oxford, Oxford, United Kingdom; ^4^ Institute of Virology, Philipps University Marburg, Marburg, Germany; ^5^ Center for Training and Research on Priority Diseases including Malaria in Guinea, Conakry, Guinea; ^6^ Department of Research, Ministry of Health Guinea, Conakry, Guinea; ^7^ Department of Virology, Bernhard Nocht Institute for Tropical Medicine, Hamburg, Germany; ^8^ School of Biological Sciences, Faculty of Environmental and Life Sciences, University of Southampton, Southampton, United Kingdom; ^9^ Department of Biochemical Sciences, School of Biosciences and Medicine, University of Surrey, Surrey, United Kingdom; ^10^ Viral Haemorrhagic Fever Reference Department, Projet Laboratoire Fièvres Hémorragiques, Conakry, Guinea

**Keywords:** complement system, immunology, virology, neutralisation, ebola virus, antibodies, protection, pathogenesis

## Abstract

The 2013–2016 Ebola virus (EBOV) epidemic in West Africa was unprecedented in case numbers and fatalities, and sporadic outbreaks continue to arise. Antibodies to the EBOV glycoprotein (GP) are strongly associated with survival and their use in immunotherapy is often initially based on their performance in neutralisation assays. Other immune effector functions also contribute to EBOV protection but are more complex to measure. Their interactions with the complement system in particular are comparatively under-researched and commonly excluded from cellular immunoassays. Using EBOV convalescent plasma samples from the 2013–2016 epidemic, we investigated antibody and complement-mediated neutralisation and how these interactions can influence immunity in response to EBOV-GP and its secreted form (EBOV-sGP). We defined two cohorts: one with low-neutralising titres in relation to EBOV-GP IgG titres (LN cohort) and the other with a direct linear relationship between neutralisation and EBOV-GP IgG titres (N cohort). Using flow cytometry antibody-dependent complement deposition (ADCD) assays, we found that the LN cohort was equally efficient at mediating ADCD in response to the EBOV-GP but was significantly lower in response to the EBOV-sGP, compared to the N cohort. Using wild-type EBOV neutralisation assays with a cohort of the LN plasma, we observed a significant increase in neutralisation associated with the addition of pooled human plasma as a source of complement. Flow cytometry ADCD was also applied using the GP of the highly virulent Sudan virus (SUDV) of the *Sudan ebolavirus* species. There are no licensed vaccines or therapeutics against SUDV and it overlaps in endemicity with EBOV. We found that the LN plasma was significantly less efficient at cross-reacting and mediating ADCD. Overall, we found a differential response in ADCD between LN and N plasma in response to various *Ebolavirus* glycoproteins, and that these interactions could significantly improve EBOV neutralisation for selected LN plasma samples. Preservation of the complement system in immunoassays could augment our understanding of neutralisation and thus protection against infection

## Introduction

Since the 2013–2016 *Zaire ebolavirus* (EBOV) epidemic in West Africa, outbreaks have continued to arise in Guinea and the Democratic Republic of the Congo (DRC), including the second largest on record in eastern DRC during 2018 which affected over 3,000 people. Protection against EBOV infection is strongly associated with the presence of anti-EBOV-GP neutralising antibodies and this knowledge has supported the development of animal models, vaccines, and therapeutics (1–5). The research efforts in this field have contributed to the FDA licensure of the Ervebo^®^ vaccine (6), EMA marketing authorisation and use of a two-dose heterologous vaccine regimen of Ad26.ZEBOV (Zabdeno^®^) boosted with MVA-BN-Filo (Mvabea^®^) (7, 8), and two licensed antibody treatments against EBOV (9). However, the emergence of new variants puts pressure on developing new interventions particularly with the current use of monoclonal antibody treatments, and other highly-virulent *Ebolaviruses* such as Sudan virus (SUDV) and Bundibugyo virus (BDBV) currently have no licensed therapeutics. A clearer understanding of what determines protection can expedite this process. Much of our current knowledge of neutralising antibodies is first based on their performance in immunoassays, but this method neglects the wider interactions with other aspects of immunity known to influence EBOV pathogenesis, such as the complement system.

**Figure 1:**
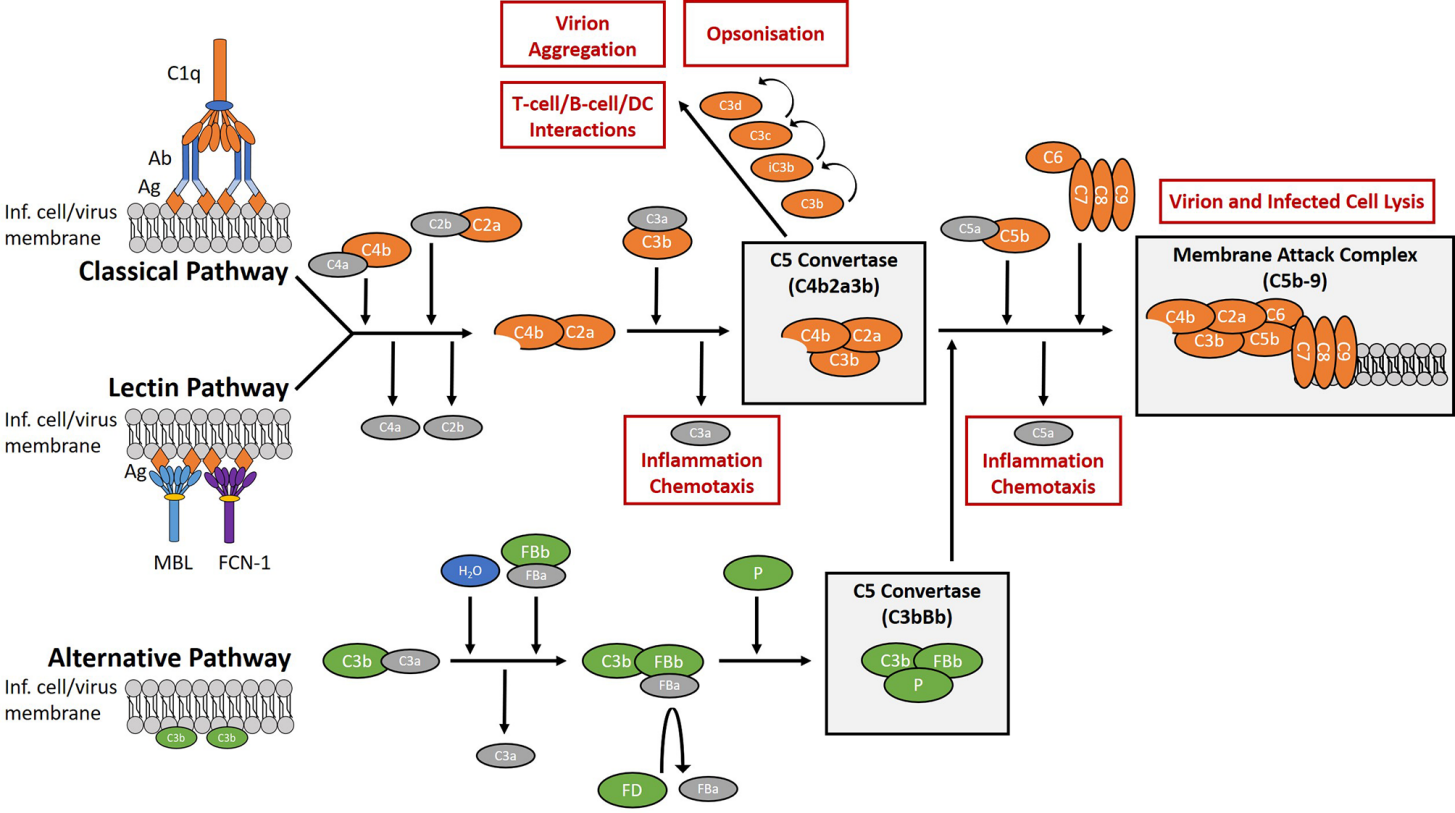
Overview of the complement system. The complement system is a collection of plasma and membrane-bound proteins which form part of the innate immune response against invading pathogens as well as performing other immunological roles. The system can be divided into three pathways (classical, lectin, alternative). The classical pathway is typically antibody-dependent and relies on the binding of C1q protein. The lectin pathway is antibody-independent and utilises lectins such as mannose-binding lectin (MBL) and ficolin-1 (FCN-1) to bind glycosylated regions on the surface antigens of pathogens. The alternative pathway is activated from spontaneous hydrolysis of the C3 protein and an absence of regulatory proteins on the microbial surface. In the context of viral infection, complement activation aims to limit infection through the promotion of inflammation and chemotaxis, the opsonisation of virions, the aggregation of virions, the direct lysis of virion and infected cell membranes, and by aiding the development of the adaptive immune response. (Ab), antibody; (Ag), antigen; (DC), dendritic cells; (FB), factor B; (FD), factor D; (FCN-1), ficolin-1; (Inf.), infected; (MBL), mannose-binding lectin; (P), properdin.

The complement system is a network of plasma and membrane-bound proteins that can be divided into three pathways (classical, lectin, alternative) which converge at a single point; the cleavage of the C3 protein (**Figure 1**). The classical pathway typically requires IgM and/or IgG in complex with the target antigen for C1q binding and pathway activation to occur. The lectin pathway is activated by the interaction of lectins with glycosylated regions of foreign antigens in an antibody-independent manner. The alternative pathway is spontaneously activated through the cleavage of C3 and primarily works to augment the lectin and classical pathways (10).

Several studies have reported on the effects of the complement system and EBOV infection. Mannose-binding lectin (MBL) of the lectin pathway has been shown to bind the EBOV-glycoprotein (EBOV-GP), resulting in the cleavage of C4 and inhibition of viral interactions with host receptor DC-SIGN *in vitro* (11). *In vivo*, recombinant MBL treatment rescued 40% of mice infected with a lethal dose of mouse-adapted EBOV (12). However, *in vitro* studies using relatively high concentrations of MBL compared to other complement proteins (13), or ficolin-1 (14), another activator of the lectin pathway, showed an enhancement of EBOV infection into various cell lines including human monocyte-derived macrophages. C1q of the classical pathway has previously been described as a possible mediator of antibody-dependent enhancement (ADE) in EBOV infections, where four distinct epitopes on the EBOV-GP were shown to mediate ADE with the use of monoclonal antibodies and C1q (15, 16), although there have been no reports of ADE following the use of the rVSV-based vaccine Ervebo^®^. Lastly, some anti-EBOV-GP monoclonal antibodies were shown to only be capable of virus neutralisation in the presence of complement (17). Furthermore, the administration of monoclonal antibodies as therapeutics in mice showed that complete protection against EBOV favoured the more efficient complement-activating murine IgG2a subclass over IgG1 or IgG3 (17). For other pathogenic *Ebolaviruses*, one report shows the risk of hearing loss in long-term sequelae post-BDBV infection is reduced with antibodies mediating antibody-dependent complement deposition (ADCD) and other polyfunctional responses (18). To our knowledge, there are currently no reports on the interactions with, or the effects of, the complement system with SUDV.

The extent of classical pathway activation in response to pathogens is known to significantly vary between antibodies, depending on factors such as antibody isotype (19–21) and epitope diversity (22–24). Common practices in handling plasma prior to use in immunoassays such as heat-inactivation or EDTA treatment inactivate the complement system and so this aspect of immunity is commonly overlooked (25–29). The importance of antibody-mediated immune effector functions independent from neutralisation have been shown for EBOV infection (30, 31) and polyfunctional immunity strongly correlates with protection (32, 33). Engineering of Fc variants has shown that the ability to mediate ADCD was crucial for complete protection against EBOV infection in an *in vivo* mouse model (34). The role of complement in EBOV infection is complex and ADCD has implications for pathogenesis and protection, yet remains largely under-researched.

In this study we assess the potential of low-neutralising plasma to engage the complement system as a possible factor in EBOV immunity. We first identified a cohort with low-neutralisation titres in relation to EBOV-GP IgG titres determined *via* ELISA (LN cohort) and another with a direct linear relationship between neutralisation and EBOV-GP IgG titres (N cohort) to reflect low-neutralising plasma and a control set of plasma, respectively. We used flow cytometry assays to determine the relationship of IgG and ADCD for LN and N EBOV convalescent plasma samples in response to EBOV-GP and its secreted form (EBOV-sGP). To demonstrate a functional effect of the LN plasma with complement, a sub-set of this cohort was used in wild-type EBOV (Makona variant) neutralisation assays supplemented with pooled human plasma (PHP) as a source of complement. Lastly, we adapted these flow cytometry methods to determine the extent of ADCD following cross-reactivity with SUDV-GP. This virus is the second-most virulent species of the *Ebolavirus* genus that infects humans and has overlapping endemicity to EBOV, yet remains comparatively under-researched.

## Methods

### Sample Collection and Identification

West African plasma samples (n = 206) were obtained as part of a longitudinal study (2015–2017) from survivors of the 2013–2016 EBOV outbreak and from EBOV negative individuals within the same region who did not come into contact with EBOV patients nor show any symptoms of EBOV disease (EVD) (35). Data collected in this longitudinal study included wild-type EBOV neutralisation assays and anti-EBOV-GP IgG ELISAs, which were correlated using 145 plasma samples available from the year 2017. From this 2017 data set we identified the LN cohort using a maximum neutralisation score cut-off of 130 geometric mean titre (GMT) of four replicates, a minimum antibody titre cut-off of 0.35 optical density (O.D.) at 405 nm, and a maximum residual cut-off from the line of best fit of -100 GMT. The N cohort was selected using a neutralisation titre cut-off greater than 200 GMT and the closest possible residual to the line of best fit to obtain matching cohort numbers. Two additional plasma samples for each cohort were identified using 2017 historical data collected prior to this study and the flow cytometry assays used within this study. Correlations were defined as follows: no correlation (R^2^ = < 0.200 and P value > 0.050), weak correlation (R^2^ = 0.210–0.400 and P value < 0.050), moderate correlation (R^2^ = 0.410–0.700 and P value < 0.050), strong correlation (R^2^ = 0.710–1.000 and P value < 0.050).

Ethical approval was obtained from the National Ethics Committee for Health Research, Guinea (33/CNERS/15) and from the National Research Ethics Service, UK for the collection and use of West African EBOV negative and convalescent plasma. All volunteers were informed of the purpose and procedures of the study and only consenting participants were included. PHP anti-coagulated with hirudin to preserve complement activity was collected from volunteers in the UK as previously described (36) and used as the exogenous source of complement for the flow cytometry and neutralisation assays described in this study.

### Protein Conjugation to Fluorescent Beads

EBOV-GP (Makona strain sourced from Nuffield Department of Medicine, Oxford University, Oxford, UK. GenBank Accession: AHX24649.1) (35), EBOV-sGP (Mayinga strain sourced from IBT Bioservices. GenBank Accession: AHC70242.1), and SUDV-GP (Gulu strain sourced from SinoBiological. GenBank Accession: YP_138523.1) proteins expressed in HEK 293 cells were covalently coupled to SPHERO™ Magnetic Flow Cytometry Multiplex Bead Assay Particles (Spherotech) at saturation levels using a modification of a previously established protocol (37). Modifications were the substitution of centrifugation steps for magnetic bead retention with the EasyEights™ EasySep™ Magnet (STEMCELL Technologies) and blocking with phosphate-buffered saline (PBS) solution containing 2% Bovine Serum Albumin (BSA), and 0.05% sodium azide (pH 7.4). Successful conjugation was determined *via* IgG detection with a known positive EBOV convalescent sample.

### Flow Cytometry Data Acquisition

For all flow cytometry experiments, samples were analysed as previously described (38), with the additional use of the PE channel for IgG, C1q, and C5b-9 detection. The gating method is demonstrated in **Supplementary Figure 1**. All samples were acquired with a CytoFLEX S flow cytometer (Beckman Coulter) collecting a minimum of 100 beads per sample, analysed using FlowJo software (version 10.8.0.), and presented using GraphPad software (Version 9).

### Flow Cytometry IgG Binding Assays

Heat-inactivated plasma (heat block at 56°C for 30 min) was diluted 1:50 in blocking buffer (Hank’s Balanced Salt Solution (HBSS), 2% BSA) for a final volume of 40 µl and titrated 1:2 for a 3-point dilution series. The final plasma dilutions (1:100, 1:500, 1:2500) were made by adding 20 µl of the EBOV-GP, EBOV-sGP, or SUDV-GP conjugated beads (50 beads per µl) into each plasma dilution. Samples were incubated for 1 h at RT whilst shaking at 550 rpm, then washed twice in 200 µl of wash buffer (HBSS, 0.05% tween-20) and resuspended in 100 µl (0.5 µg/ml) PE-conjugated anti-human IgG (Cambridge Bioscience) in blocking buffer. Samples were again incubated for 1 h at RT whilst shaking at 550 rpm, washed twice in 200 µl of wash buffer, and resuspended in 50 µl HBSS.

For all IgG assays, three plasma dilutions with the EBOV-GP conjugated beads were used as quality controls (QCs) for assay performance and were all below 30% CV (**Supplementary Figure 3A**). Further controls were included for the SUDV-GP and EBOV-sGP assays using their respective bead conjugates to monitor the bead integrity at a single dilution point (**Supplementary Figure 3B**) and were all below 15% CV. The final results were reported using a single dilution point which avoided assay saturation and subtracted the relevant negative plasma sample from each plate.

### Flow Cytometry C1q Binding Assay

Each bead conjugate (1000 beads per sample) was incubated with heat-inactivated EBOV survivor plasma with known IgG binding to EBOV-GP, EBOV-sGP, and SUDV-GP, along with anti-EBOV-GP negative plasma, at a final 1:20 plasma dilution. The bead and plasma mixture was then incubated for 30 min at 25°C whilst shaking at 900 rpm, washed twice in 200 µl of wash buffer, and resuspended in 100 µl of purified C1q protein (Sigma Aldrich) in blocking buffer at 5 µg/ml, 2.5 µg/ml, 1.25 µg/ml or with blocking buffer only. The samples were then incubated at 25°C for 1 h whilst shaking at 900 rpm, washed twice in 200 µl of wash buffer, resuspended in 100 µl (1 µg/ml) anti-C1q monoclonal antibody (Quidel) and incubated at 25°C for 30 min whilst shaking at 900 rpm. The samples were washed again, resuspended in 100 µl (1 µg/ml) PE-anti-mouse IgG (ThermoFisher Scientific) and incubated at 25°C for 30 min whilst shaking at 900 rpm. The final wash step was carried out and the samples resuspended in 50 µl HBSS. A negative cut-off was determined using an average of all bead and plasma controls which excluded the primary antibody step, plus three standard deviations.

### Flow Cytometry C3c and C5b-9 Deposition Assays

The methods used in this study have previously been utilised and published for detecting antibody-dependent C3c deposition on the SARS-CoV-2 spike protein (38) and used again in a current preprint (39). EBOV-GP, EBOV-sGP, or SUDV-GP conjugated beads (50 beads per µl) were mixed with heat-inactivated EBOV survivor plasma diluted four times at a 1:2 dilution starting from 1:10 (SUDV-GP) or 1:20 (EBOV-GP and EBOV-sGP) and incubated for 30 min at 25°C whilst shaking at 900 rpm. The beads were washed twice with 200 µl wash buffer and resuspended in 50 µl PHP (diluted 1:10 in blocking buffer) as a source of complement, then incubated for 15 min at 37°C with shaking at 900 rpm. For C3c detection, the beads were washed twice with 200 µl wash buffer and resuspended in 100 µl FITC-conjugated rabbit anti-human C3c polyclonal antibody (Abcam) diluted 1:500 in blocking buffer and incubated for 20 min in the dark. For detection of C5b-9 deposition, the C3c antibody was replaced with a monoclonal C5b-9 antibody (SantaCruz Biotechnology) at a concentration of 1 µg/ml in 100 µl blocking buffer. A further wash step and incubation with 100 µl PE-conjugated anti-mouse polyclonal antibody (ThermoFisher Scientific) at 1 µg/ml in blocking buffer for 20 min in the dark was required for C5b-9 detection. For both C3c and C5b-9 deposition assays, the beads were washed a final two times in 200 µl wash buffer before re-suspension in 50 µl HBSS.

Each test plate included a heat-inactivated negative plasma control (EBOV naïve Guinean plasma sample), a PHP-only control, a conjugate-only control, a plate QC using EBOV-GP beads with a fixed plasma dilution (C3c: **Supplementary Figure 4A**, C5b-9: **Supplementary Figure 5A**), and a bead QC using either EBOV-GP, EBOV-sGP, or SUDV-GP beads at a fixed plasma dilution (C3c: **Supplementary Figure 4B**, C5b-9: **Supplementary Figure 5B**). All plate and bead QCs were within 30% CV. Where the dilutions for some samples saturated the assay, linear regression was used from larger dilutions to predict these values. Where multiple bead batches were used (EBOV-GP C3c assay), the negative sample median fluorescence intensity (MFI) on each plate was subtracted from all samples to best normalise the data based on the QCs. All other assays used a single bead batch where the PHP-only control was subtracted.

### Wild-Type EBOV Neutralisation Assay

Wild-type EBOV neutralisation assays were performed in the BSL4 laboratory at the Institute of Virology, Philipps University of Marburg, Germany. Virus neutralisation assays were a modification of methods previously described using the EBOV Makona variant (40, 41). Briefly, eight plasma samples randomly selected from the LN cohort and one high neutralising control sample were serially diluted 1/2^3^ to 1/2^8^ in 50 µl Dulbecco’s modified Eagle’s medium (DMEM) supplemented with penicillin (100 U/ml), streptomycin (100 mg/ml), L-glutamine (2 mmol/l), and exogenous PHP for a final concentration of 20%, 10% or 0%. 100 TCID50 units (50 µl) of EBOV (Makona isolate, GenBank accession No. KJ660347) in DMEM with 2% FCS were added to the plasma dilutions and incubated at 37°C for 1 h. Vero cells diluted in DMEM containing 2% FCS (9.4x10^3^ cells) were then added to each well and the plates were incubated at 37°C with 5% CO_2_ and cytopathic effects analysed at nine days post infection. Neutralisation titres were calculated as geometric mean titres of four replicates. Each plate included PHP-only, cell-only, and heat-inactivated PHP controls.

Individual samples were analysed *via* a log2 fold-change of GMT compared to the plasma-only condition with a significance threshold of +/- 1.5. Each group (plasma-only, 10% PHP, 20% PHP) was compared in a pairwise manner using a one-tailed, Wilcoxon signed-rank test as an increase was expected with PHP, and a significance threshold of P < 0.050. Samples were analysed and presented using GraphPad (Version 9).

## Results

### Selection of Low-Neutralising Plasma Samples and Confirmation of IgG/C1q Binding

Low-neutralising plasma samples against EBOV were of particular interest in this study as we aimed to characterise their possible interaction with the complement system and whether this interaction enhanced neutralisation. Previous studies successfully demonstrating a complement-mediated enhancement of antibody neutralisation were performed using non or low-neutralising antibodies (17, 42–44) and so the same hypothesis was applied here.

**Figure 2:**
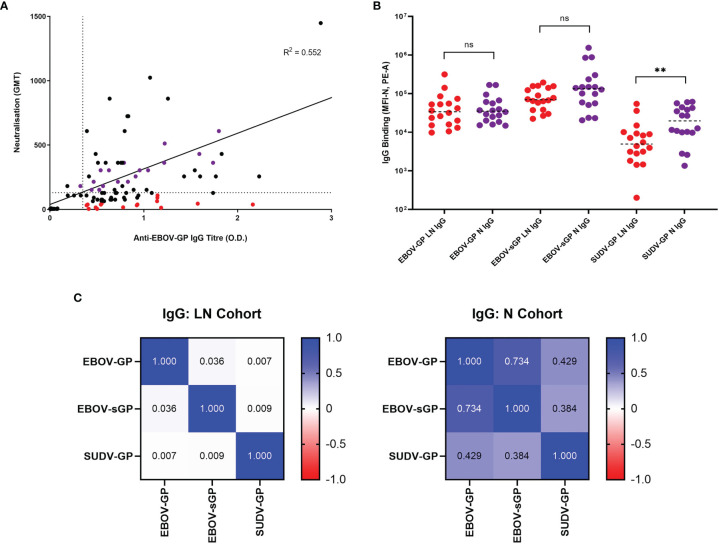
Selection of EBOV-GP plasma samples and their abilities to bind EBOV-GP, EBOV-sGP, and SUDV-GP. **(A)** 145 samples from historic EBOV-GP ELISAs and EBOV neutralisation assays were correlated and analysed *via* linear regression. Samples greater than the axis limits were excluded from the graph for the purpose of clarity, but still included in the analysis. The LN cohort (red dots, n = 16) was selected using a neutralisation cut-off < 130 GMT (horizontal dotted line) and an antibody titre > 0.35 O.D. (vertical dotted line), with a maximum residual from the line of best fit (< -100 GMT). The N cohort (purple dots, n = 16) was selected using a neutralisation cut-off > 200 GMT and the closest possible residual to the line of best fit. **(B)** EBOV-GP, EBOV-sGP, and SUDV-GP conjugated beads were incubated with plasma from the LN (n = 18) or N (n = 18) cohorts and analysed *via* flow cytometry. Mean values are represented by horizontal dotted lines and significance determined using a Mann-Whitney U test. **(C)** A pairwise linear regression analysis was performed for each bead conjugate with LN (n = 18) and N (n = 18) plasma cohorts and the R^2^ values for IgG binding were presented in the form of a heatmap. ** = significant (P < 0.01), ns, Not significant.

Analysis of the correlation between historic anti-EBOV-GP IgG and neutralisation data (35) (**Figure 2A**) showed a similar distinction of cohorts to anti-EBOV-GP IgG determined *via* flow cytometry in this study (including four additional plasma samples) and the same historic neutralisation data (**Supplementary Figure 2**). Using flow cytometry, we observed IgG binding to EBOV-GP (**Supplementary Figure 3C**), EBOV-sGP (**Supplementary Figure 3D**), and SUDV-GP (**Supplementary Figure 3E**) with all convalescent plasma samples. No binding was observed when using EBOV negative plasma. These proteins belong to the two most virulent species of the *Ebolavirus* genus and were selected as they are presented on the surface of the virions and infected cells or are actively secreted into the extracellular space. Therefore, they are the most likely EBOV proteins to encounter the complement system, where the first step in conventional activation of the classical pathway is the binding of IgG.

After subtracting the MFI of the negative plasma sample on each plate from all samples at the chosen dilutions, the total IgG binding of the LN and N cohort was compared for each protein (**Figure 2B**). For EBOV-GP and EBOV-sGP, the overall IgG titres showed no significant difference when using a Mann-Whitney U test (P < 0.050). For SUDV-GP, the N cohort demonstrated a significantly higher level of IgG binding compared to the LN cohort (P = 0.005) with a 1.4 log2 fold increase. The final MFI for IgG binding of the LN and N cohorts to each protein were then analysed *via* linear regression (**Figure 2C**). Differences in the relationship of IgG binding to *Ebolavirus* proteins for each cohort might indicate variations in antibody epitopes, antibody diversity, or cross-reactivity, which are important factors for ADCD. For the N cohort, there was a strong correlation between EBOV-GP and EBOV-sGP (R^2^ = 0.734), a moderate correlation between SUDV-GP and EBOV-GP (R^2^ = 0.429) and a weak correlation between SUDV-GP and EBOV-sGP (R^2^ = 0.384). For the LN cohort, there was no correlation between any of the proteins tested: EBOV-GP and EBOV-sGP (R^2^ = 0.036), SUDV-GP and EBOV-GP (R^2^ = 0.007), and SUDV-GP and EBOV-sGP (R^2^ = 0.009).

**Figure 3:**
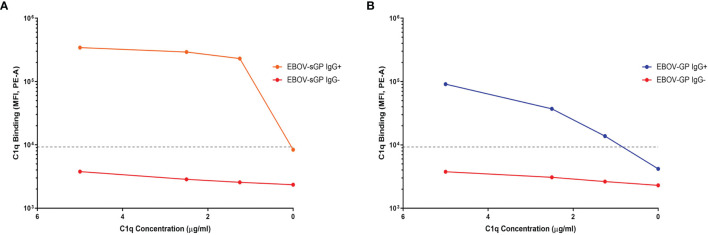
The binding of purified C1q protein to EBOV-GP and EBOV-sGP in antibody complexes. Purified C1q protein was titrated against EBOV-GP **(A)** and EBOV-sGP **(B)** conjugated beads with human plasma in the presence or absence of EBOV-GP IgG. A negative cut-off (grey dotted line) was determined using the mean value for all control samples where the primary antibody was excluded, plus three standard deviations.

Following antibody binding, the next step in the activation cascade of the classical complement pathway would typically be the binding of the C1q protein to IgG/IgM in complex with the target antigen. In this study, the detection of C1q binding was only observed following addition of purified C1q to plasma containing anti-EBOV-GP IgG bound to EBOV-GP or EBOV-sGP. We did not observe C1q binding in the presence of anti-EBOV-GP IgG negative plasma nor with the use of SUDV-GP conjugated beads (**Figure 3** and **Supplementary Figure 6**). C1q binding may occur in the absence of antibodies by directly binding the antigen or utilising acute phase proteins against some pathogens. However, our results indicate that C1q binding to EBOV-GP and EBOV-sGP was dependent on the presence of IgG.

In summary, we identified plasma from two cohorts based on their ability to neutralise EBOV relative to their anti-EBOV-GP IgG titres. The LN cohort showed significantly lower IgG binding to the SUDV-GP compared to the N cohort despite similar anti-EBOV-GP and anti-EBOV-sGP titres. Furthermore, the LN cohort showed no clear relationship in IgG titres between the *Ebolavirus* proteins, whereas IgG binding in the N cohort correlated as expected (**Figure 2**). C1q binding could be detected following the binding of IgG to EBOV-GP and EBOV-sGP (**Figure 3**).

### ADCD and Its Relationship With IgG Binding

After confirmation of IgG and C1q binding, the extent of ADCD *via* the classical pathway for the N and LN cohort was determined by the levels of C3c and C5b-9 deposition. Complement activation can have both local and systemic effects on a wide range of immune functions. The extent of this activation varies depending on antibody characteristics and so provides a mechanism through which LN plasma samples could influence EBOV pathogenesis.

**Figure 4:**
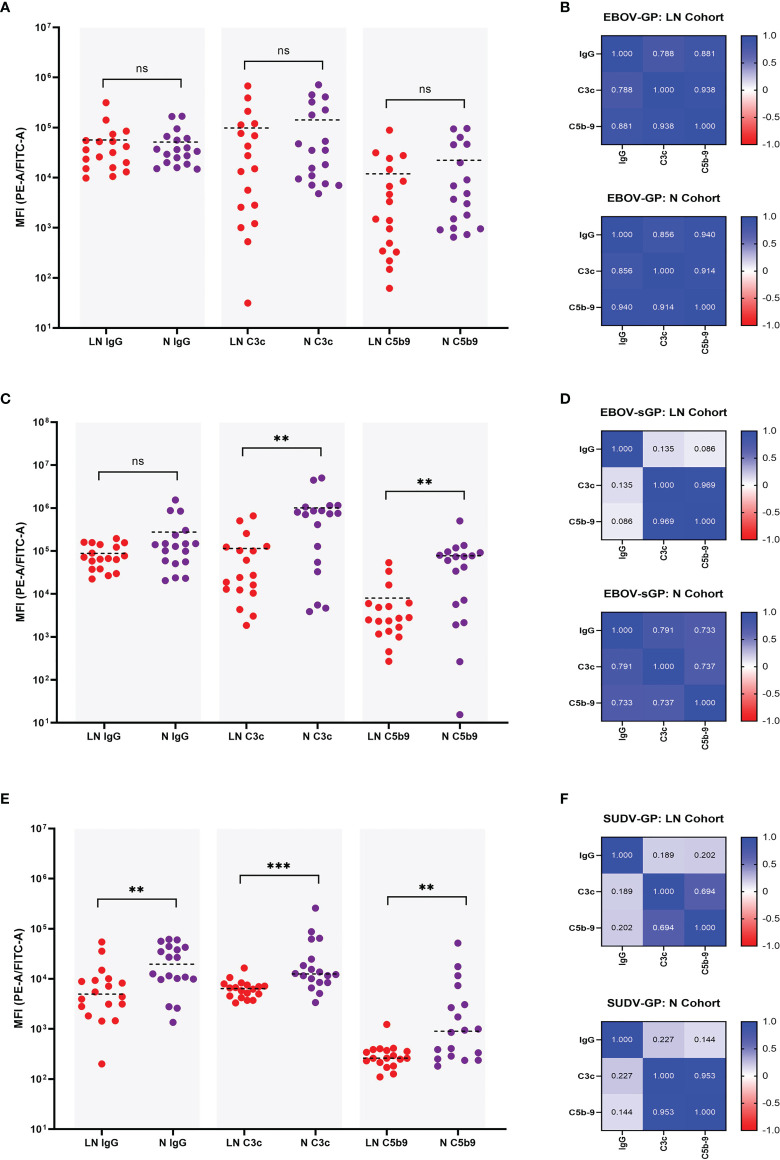
Comparison of IgG binding, C3c deposition, and C5b-9 deposition for EBOV-GP, EBOV-sGP, and SUDV-GP. Plasma samples in the N cohort (purple dots, n = 18) and LN cohort (red dots, n = 18) were compared using a Mann-Whitney U test for IgG binding, C3c deposition and C5b-9 deposition against EBOV-GP **(A)**, EBOV-sGP **(C)**, and SUDV-GP **(E)**. The pairwise relationship between each parameter for both cohorts was then analysed *via* linear regression and the R^2^ values reported as a heatmap for EBOV-GP **(B)**, EBOV-sGP **(D)**, and SUDV-GP **(F)**. Grey shaded areas group the samples based on assay type. ** = significant (P < 0.01), *** = significant (P < 0.001), ns, not significant.

For the EBOV-GP LN and N cohort there was no significant difference in IgG binding (P = 0.673) (previously shown in **Figure 2B**), C3c deposition (P = 0.239), nor C5b-9 deposition (P = 0.181) using a Mann-Whitney U test (**Figure 4A**). A linear regression analysis was also performed to assess the relationship between these three parameters and the R^2^ values presented as a heatmap in **Figure 4B**. Both the N and LN cohort showed a strong correlation for C3c and C5b-9 deposition with R^2^ = 0.914 and R^2^ = 0.938, respectively. IgG values also strongly correlated with C3c for the N cohort (R^2^ = 0.856) and the LN cohort (R^2^ = 0.788), and again with C5b-9 for the N (R^2^ = 0.940) and LN (R^2^ = 0.881) cohorts.

EBOV-sGP total IgG binding showed no significant difference (P = 0.239) between the LN and N cohorts (previously shown in **Figure 2B**, yet the N cohort showed significantly higher levels of C3c deposition (P = 0.002) and C5b-9 deposition (P = 0.003) (**Figure 4C**). The linear regression analysis (**Figure 4D**) showed a strong correlation when analysing the N cohort and LN cohorts for C3c and C5b-9 deposition of R^2^ = 0.737 and R^2^ = 0.969, respectively. For C3c and IgG, a strong correlation was observed for the N cohort (R^2^ = 0.791), but no correlation was observed for the LN cohort (R^2^ = 0.135). Similar findings were observed for IgG and C5b-9 deposition, with a strong correlation for the N cohort (R^2^ = 0.733) but no relationship with the LN cohort (R^2^ = 0.086).

For SUDV-GP, the N cohort showed significantly higher IgG binding (P = 0.005) (previously shown in **Figure 2B**), C3c deposition (P = < 0.001), and C5b-9 deposition (P = 0.004) (**Figure 4E**) compared to the LN cohort. The linear regression analysis (**Figure 4F**) showed a strong correlation for C3c and C5b-9 deposition for the N cohort (R^2^ = 0.953) and a moderate correlation for the LN cohort (R^2^ = 0.694). For C3c and IgG, a weak correlation was observed for the N cohort (R^2^ = 0.227) but no correlation for the LN cohort (R^2^ = 0.189), and no correlation was observed for IgG and C5b-9 deposition for the N cohort (R^2^ = 0.144) or the LN cohort (R^2^ = 0.202).

In summary, the levels of ADCD varied depending on whether the plasma was from the LN or N cohort, and depended on the *Ebolavirus* protein present (**Figure 4**). For EBOV-GP, both plasma cohorts showed similar levels of ADCD and this response correlated strongly with IgG titres. For EBOV-sGP, the LN cohort was less efficient at mediating ADCD despite similar IgG binding titres to the N cohort. Whilst ADCD with the N cohort appeared dependent on IgG titre, ADCD and IgG titre did not correlate for the LN cohort. Results using SUDV-GP were also different, as the LN cohort was significantly lower than the N cohort for all parameters tested, and ADCD in neither cohort correlated with IgG titre.

### The Effect of PHP on Wild-Type EBOV Antibody Neutralisation Assays

As mentioned previously, antibodies can activate the complement system and influence a number of immune effects, both local and systemic. One of these effects is the enhancement or development of neutralisation in low or non-neutralising antibodies, respectively. Since the LN cohort demonstrated a clear ability to mediate ADCD, we investigated the effect on wild-type EBOV neutralisation with the addition of exogenous PHP.

**Figure 5:**
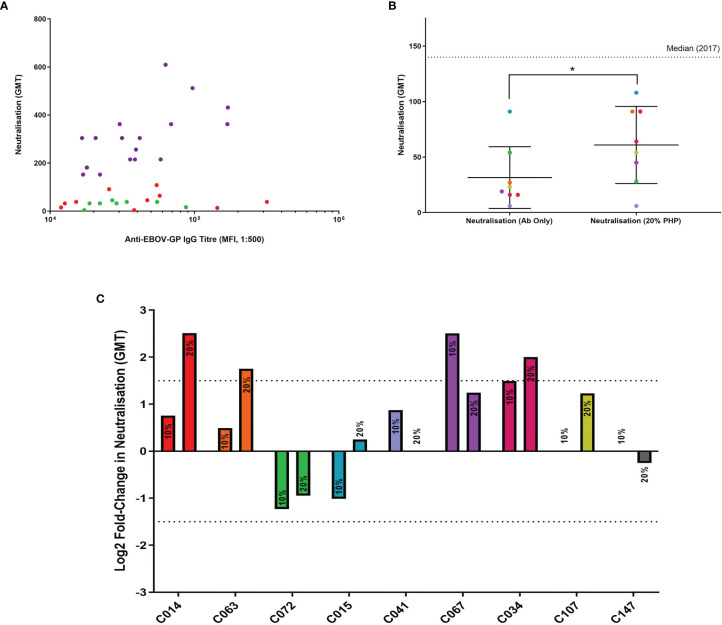
Antibody neutralisation assays with wild-type EBOV, supplemented with PHP as a source of complement. **(A)** Eight samples from the LN cohort were selected for use in wild-type EBOV neutralisation assays (green dots). The remaining samples represent the LN cohort (red dots) and the N cohort (purple dots). All samples are illustrated using IgG titres determined in this study *via* flow cytometry and compared to historic 2017 neutralisation data. **(B)** Comparison of LN cohort neutralisation with or without 20% PHP, analysed using a one-tailed Wilcoxon signed-rank test (P = 0.031). **(C)** Comparison of individual samples analysed *via* a log2 fold-change with 10% and 20% PHP compared to antibody-only samples, with a negative cut-off below 1.5 (dotted line). * = significant (P < 0.05).

Eight samples from the LN cohort were selected at random (**Figure 5A**) for wild-type EBOV neutralisation assays with or without the addition of exogenous PHP, and a strongly neutralising positive control (sample C147). The antibody-with-PHP group had a significantly higher (P = 0.031) neutralisation titre than the antibody-only group as part of the same assay (**Figure 5B**). The significance was also higher when comparing the antibody-with-PHP group to historic data from the 2017 assay (P = 0.012), whilst the antibody-only groups in this study and from 2017 showed no significant difference (P = 0.500). The addition of PHP at 20% resulted in a significant increase but remains below the median value of the 132 survivor samples tested in the historic 2017 cohort (35).

When the log2 fold change for each sample was analysed with 10% and 20% PHP compared to their antibody-only controls, one sample showed a significant increase in neutralisation with 10% PHP (CO67) and three samples significantly increased when using 20% PHP (**Figure 5C**). No samples showed a significant decrease in neutralisation with the addition of PHP. No cytotoxic effects were observed when using PHP at 10% and 20% concentrations. However, when increasing PHP to 40%, evidence of cell cytotoxicity emerged and this data was subsequently excluded from the study (**Supplementary Figure 7**).

In summary, the addition of PHP as a source of complement was able to significantly increase the level of neutralisation for selected LN plasma samples. This effect was most noticeable with the highest PHP concentration tested at 20%.

## Discussion

In this study we describe a potential role of the complement system in the context of *Ebolavirus* infections mediated by convalescent EBOV plasma. The use of LN plasma expands on our current knowledge regarding Fc-mediated EBOV antibody functions. First, we demonstrated the potential for convalescent EBOV plasma samples to mediate ADCD and showed that this response significantly varied depending on whether the samples were from the LN or N cohort. Furthermore, ADCD was dependent on the type of EBOV protein present in the assay (EBOV-GP, EBOV-sGP, or SUDV-GP), indicating a possible function in *Ebolavirus* cross-reactivity. These interactions resulted in a significant enhancement of neutralisation for selected samples when tested against wild-type EBOV. These findings show a capacity for LN antibodies to mediate ADCD in the context of EBOV infection, influencing neutralisation and potentially further local and systemic immune responses including pro-inflammatory responses, chemotaxis, opsonisation, agglutination, and immune cell regulation. This highlights an acute need for further research to fully determine the role of these mechanisms in immunity.

We found that plasma samples from the LN cohort were less capable of cross-reacting with SUDV-GP and that there was no relationship of IgG binding between all proteins, unlike the N cohort (**Figure 2**). The significantly lower level of LN IgG binding to SUDV-GP suggests that either the epitopes recognised by plasma in the N cohort are better conserved amongst these proteins, or that the N cohort plasma may have a more diverse antibody response to recognise a broader range of targets. This could also explain the lack of correlation of LN IgG binding to SUDV-GP compared with EBOV-GP and EBOV-sGP. The lack of correlation of LN plasma IgG bound to EBOV-GP and EBOV-sGP may be because different plasma antibodies target certain conformational epitopes of either the sGP or whole EBOV-GP such that overall binding is not affected (45). It’s possible that the bead conjugation process restricts certain epitopes. However, this method utilises abundant and regularly distributed free amine groups on these glycoproteins and so this is unlikely. Whilst care was taken to ensure all proteins were obtained from HEK 293 mammalian cell expression systems to reduce differences in glycosylation and protein processing, they originated from different suppliers and relied upon different methods of purification.

Activation of the classical complement pathway typically requires IgG/IgM binding prior to engagement of the C1q protein, although in rare instances C1q may bind viral antigens directly to activate the complement system (46–48). In our observations, ADCD in response to EBOV-GP and EBOV-sGP was dependent on the binding of anti-EBOV-GP antibodies (**Figure 3**). The absence of C1q:IgG binding for SUDV-GP was likely an assay sensitivity issue due to the lower levels of IgG binding. This would decrease the number of targets for the C1q protein and have a lower epitope density, thus reducing the formation of antibody clusters required for efficient C1q binding (24) and complement activation (22, 23). Based on previous studies regarding antibody kinetics (49, 50), we do not anticipate that these samples, collected at least 1-year post-exposure, would contain significant levels of IgM. These results highlight some important functional differences in the initial stages of ADCD between the LN and N cohorts.

We report a significant difference in the levels of ADCD between the LN and N cohorts and the relationship of deposition to IgG, depending on the EBOV protein present (**Figure 4**). For EBOV-GP (**Figures 4A, B**), the LN plasma IgG was equally efficient at binding the target protein and mediating ADCD compared to the N cohort plasma, and the level of deposition for both cohorts was dependent on IgG titre. Therefore, both LN and N plasma could play a role in EBOV infection through the activation of complement, which in turn can promote inflammation and chemotaxis (10, 51) and reduce viral load (47, 52–54).

For EBOV-sGP (**Figures 4C, D**), despite similar IgG titres, the LN cohort was significantly less efficient at mediating ADCD compared to the N cohort. This could be the result of antibody isotypes involved in binding for each cohort, as IgG1 and IgG3 activate complement most efficiently, followed by IgG2, whilst IgG4 has no activity and may even be inhibitory (19–21). Epitope density, antibody recognition, and antibody clustering for efficient C1q binding may also influence activation as discussed previously. Interestingly, and unlike the N cohort, IgG binding and ADCD did not correlate for the LN plasma. This may be explained by the isotype ratio and epitope specificity leading to varying efficiencies in complement activation. Acute-phase reactive proteins that facilitate C1q binding in place of antibodies (10) might explain this phenomenon. However, the N cohort correlated positively with IgG as expected, we did not observe C1q binding with EBOV negative plasma, and we only observed negative results for the PHP-only controls. Furthermore, the EBOV-GP did not show a similar trend which might otherwise be expected. It is possible that the difference in strains used for the EBOV-GP (Makona) and EBOV-sGP (Mayinga) might affect some of the comparisons being made, as the survivor cohort were infected with the Makona variant. It is therefore possible that antibodies targeted to a non-homologous region of the EBOV-GP could be missed when comparing binding and subsequent ADCD on the EBOV-sGP. A comparison of the full-length genomes of representative EBOV isolates shows an estimated 97% sequence identity between the Makona and Mayinga variants used in this study (55).

The sGP is the primary transcript from the EBOV *GP* gene (56) and is actively secreted during infection to levels detectable in the blood of acutely infected patients (57). The sGP is purportedly an antibody decoy molecule capable of subverting the immune response away from GP_1,2_ and inhibiting neutralisation (58, 59). Antibody-mediated complement activation in response to high levels of sGP could be an interesting focus for future studies, as complement depletion (60) and the production of decoy molecules for complement evasion (61) are disease mechanisms described for other pathogens that may be relevant to sGP. Excessive complement activation has been associated with fatal EVD outcome based on transcriptomic signatures (62) and has been shown to exacerbate other viral infections (63–67). It is therefore possible that the ADCD we describe here in response to EBOV-sGP could influence EBOV immunity.

For SUDV-GP (**Figures 4E, F**), the LN cohort had significantly lower levels of bound IgG, C3c deposition, and C5b-9 deposition compared to the N cohort. Whilst C3c and C5b-9 deposition showed a clear correlation for both cohorts, the association of IgG compared to C3c and C5b-9 deposition was either weak or not significant. As mentioned previously, the IgG isotypes and/or the antibody epitopes to enable clustering and C1q binding may account for some of this variation. The involvement of complement in cross-reactivity with SUDV from EBOV convalescent plasma could have implications for cross-protection, resulting in complement deposition, the production of C3a and C5a anaphylatoxins, and the formation of the membrane attack complex (**Figure 1**). Our results suggest the level of this response would vary depending on the capacity for neutralisation of plasma samples. However, it is not clear how these levels would translate *in vivo* and whether the resulting effects would be beneficial or detrimental to immunity.

Previous reported outbreaks of EBOV and SUDV have occurred in geographically similar areas, with EBOV causing repeated outbreaks in DRC and on one occasion a spillover into neighbouring Uganda, whilst SUDV has the been the cause of multiple outbreaks in Uganda and the neighbouring South Sudan (68). These three countries also provide suitable habitats for putative EBOV bat reservoirs based on a MaxEnt niche modelling approach (69). Furthermore, the added complexity of human-to-human transmission (70), viral persistence in immune privileged sites including ocular fluid (71), semen (72, 73), breast milk (74, 75) and cerebrospinal fluid (76), the potential for recrudescence (71, 76), and a general lack of resources for viral surveillance in the affected areas complicate the spread, transmission, and possible overlap of these viruses.

The addition of PHP to LN plasma in wild-type EBOV neutralisation assays resulted in a significant enhancement to their neutralisation (**Figure 5**). In a previous study, the presence of complement has been shown to enable neutralisation with otherwise non-neutralising purified anti-EBOV-GP antibodies (17), although the addition of complement to human plasma in a different study showed no significant difference (77). An important distinction between the latter study and ours may be the use of human instead of guinea pig complement which shows some key functional differences (78–80), or their use of historical samples 40 years after infection as the non-complement activating IgG-4 isotype reportedly starts developing 1–2 years post-EBOV infection (81). In our study, a significant increase in wild-type EBOV neutralisation was observed using 20% PHP compared to the antibody-only controls. The increase in neutralisation was considered modest as the neutralisation values remained below the median neutralisation value of all 132 survivor samples from the 2017 historic data (35). However, it should be noted that EBOV is a blood-borne pathogen and would encounter high concentrations of complement during infection. One previous study investigating the effects of Zika virus and complement from normal human serum (NHS) used concentrations up to 50% (with EDTA) with an increasingly positive trend between neutralisation and NHS concentration (47). We increased the PHP concentration to 40% to see if the trend in increasing neutralisation would continue, but evidence of cell cytotoxicity emerged (**Supplementary Figure 7**). To test these higher concentrations, the use of EDTA may be required post-virus incubation and pre-cell infection. This would preserve cell integrity but potentially overlook complement interactions with the sGP which is secreted during infection and the possible lysis of infected cells (52, 82) or prevention of spread into neighbouring cells (17).

When analysed individually, we found a subset of samples that were significantly influenced by the presence of complement (**Figure 5**). This is in agreement with similar studies (42–44, 54) and this difference has previously been attributed to the antibody subclass (17). Compared to the latter study by Wilson *et al*, our observed increase in neutralisation was lower. This may be explained by our use of native plasma which better represents the polyclonal antibody response of natural immunity compared to the use of a single purified antibody. Sample C067 showed a significant increase in neutralisation with 10% PHP but fell just below the threshold when PHP was increased to 20%. This observation could be the result of inherent assay variance as the difference falls within the 1.5 log2 fold-change threshold. It is unlikely that our observations are explained by saturation with 10% PHP, as the majority of samples show a positive trend between 10% and 20% PHP concentration and neutralisation. The different antibody repertoires between plasma samples will affect how they engage complement proteins and thus impact on immunity, however our sample size was restricted due to limited resources. These results show that the commonly used neutralisation immunoassays can be limited by excluding complement and this should be noted when considering such assays, including *in vivo* experiments or screening for therapeutics.

In conclusion, we show a potential for complement-mediated enhancement of antibody-mediated immunity in both the LN and N cohorts and highlight where they significantly differ depending on IgG binding, neutralising ability, and the *Ebolavirus* protein used. This may have implications for wider immune responses important to EVD such as inflammation and chemotaxis that could be pursued in future studies. We have also shown how LN plasma can neutralise wild-type EBOV more efficiently in the presence of complement at relatively low concentrations. Future investigations of antibody-mediated neutralisation may benefit from the addition of complement to immunoassays and should consider the use of EDTA when testing higher complement concentrations. As our findings are assumed to be IgG-mediated, they hold most relevance to re-exposure, recrudescence, vaccination, and cross-reactivity with *Ebolaviruses*. Future studies may consider similar investigations with IgM or the possibility of acute-phase reaction proteins to engage complement. Flow cytometry methods used in this study have previously been applied to the SARS-CoV-2 spike protein where multifunctional antibody responses beyond neutralisation could be important for protection (38) and similarly, investigations on variants of concern demonstrate that neutralisation as classically defined should not be considered as the sole determinant of protection (83).

## Data Availability Statement

The original contributions presented in the study are included in the article/**Supplementary Material**. Further inquiries can be directed to the corresponding authors.

## Ethics Statement

The studies involving human participants were reviewed and approved by National Ethics Committee for Health Research, Guinea (33/CNERS/15) and from the National Research Ethics Service, UK. The patients/participants provided their written informed consent to participate in this study.

## Author Contributions

Authors MC, JHi, ST, NM, and MK conceptualized the study. Plasma sample collection and processing was conducted by TT, YH, JA, FK, and FA. Authors JM, TT, TS, SKF, FA, and JHu contributed to the experimental work and authors JM, TS, SKF, and MC were involved in data analysis. Lastly, authors JM, TT, TS, SKF, ST, AG, JHu, YH, SL, and MC contributed to the writing of the manuscript.

## Funding

This work was funded via a UK Health Security Agency studentship programme and by the US Food and Drug Administration Medical Countermeasures Initiative contract 75F40120C00085 and contract HHSF223201510104C. Author T.S. received funding from the Deutsche Forschungsgemeinschaft (DFG, German Research Foundation) - Projektnummer 197785619 - SFB1021.

## Conflict of Interest

The authors declare that the research was conducted in the absence of any commercial or financial relationships that could be construed as a potential conflict of interest.

## Publisher’s Note

All claims expressed in this article are solely those of the authors and do not necessarily represent those of their affiliated organizations, or those of the publisher, the editors and the reviewers. Any product that may be evaluated in this article, or claim that may be made by its manufacturer, is not guaranteed or endorsed by the publisher.
