# Supplementary material for: Complement-Mediated Neutralisation Identified in Ebola Virus Disease Survivor Plasma: Implications for Protection and Pathogenesis
Source: Front Immunol. 2022 Apr 12;13:857481. doi: 10.3389/fimmu.2022.857481 (PMC9039621; doi:10.3389/fimmu.2022.857481)
Supplement: Supplementary file 1 [file DataSheet_1.docx]

Supplementary Material

| **(A)**  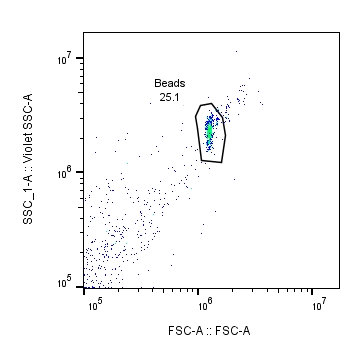 | | **(B)**  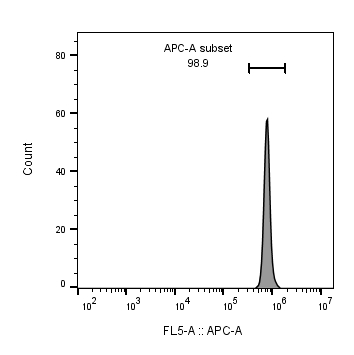 | |
| --- | --- | --- | --- |
| **(C)**  **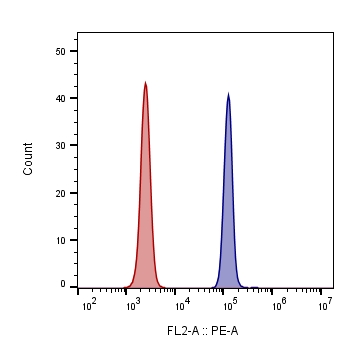** | **(D)**  **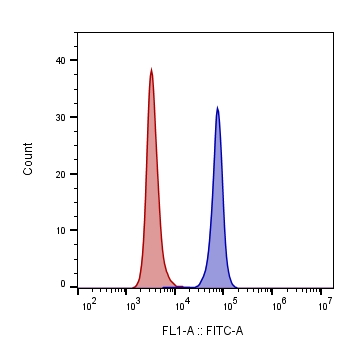** | | **(E)**  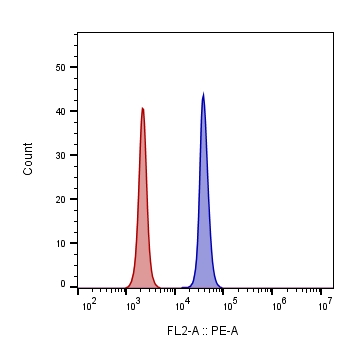 |

**Supplementary Figure 1: Gating strategy for all flow cytometry assays.**

All flow cytometry samples were analysed using FlowJO software (Version 10.8.0) to determine the median fluorescence intensity (MFI). **(A)** The first gate was created on the conjugated bead samples using the forward scatter versus the violet side scatter. **(B)** The APC channel was then used to create a second gate on the beads. MFI’s for **(C)** IgG binding, **(D)** C3c deposition, and **(E)** C5b-9 deposition were then determined using either the FITC or PE channel, illustrated with a negative (red) or positive (blue) EBOV-GP IgG plasma sample.

| 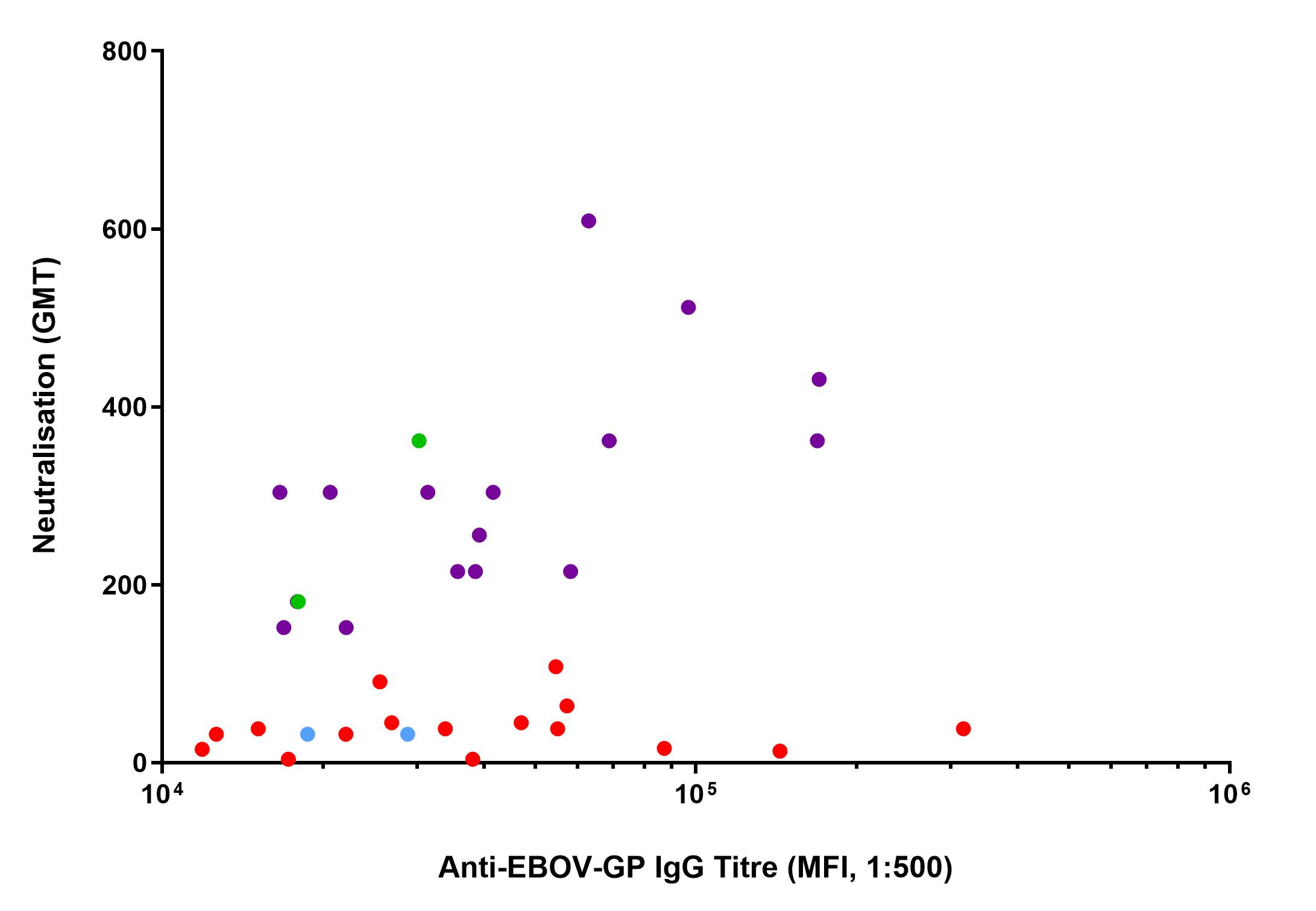 |
| --- |

**Supplementary Figure 2: Selection of additional EBOV-GP plasma samples using flow cytometry**

EBOV-GP IgG titres determined via flow cytometry in this study were correlated with historic neutralisation data from the year 2017 for all samples. The LN cohort (red dots) and N cohort (purple dots) were initially identified using historic EBOV-GP ELISA data and neutralisation data. Two additional samples for the LN cohort (blue dots) and N cohort (green dots) were absent from the historic ELISA data but later included based on the flow cytometry data in this study.

| **(A)** 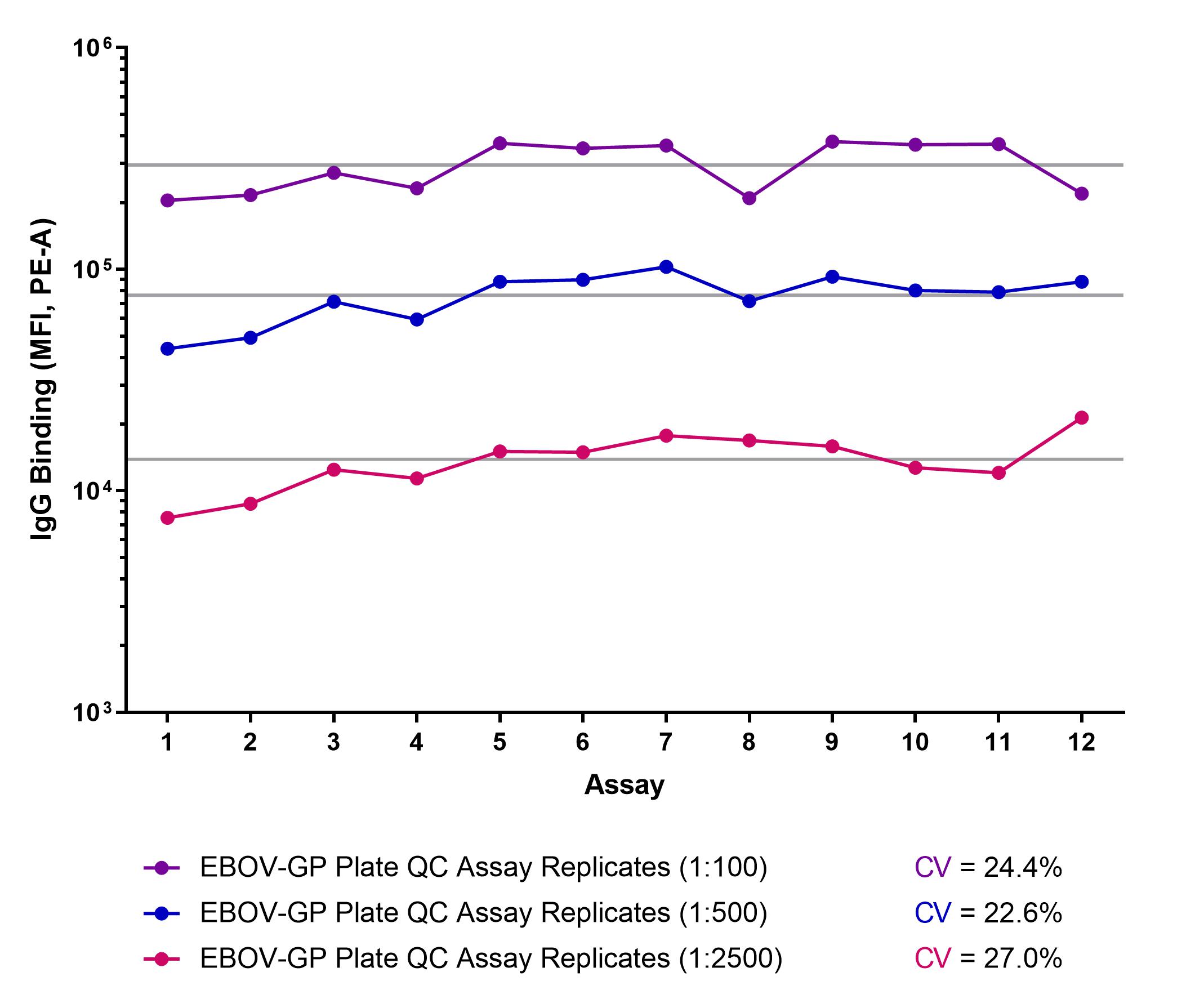 | **(B)**  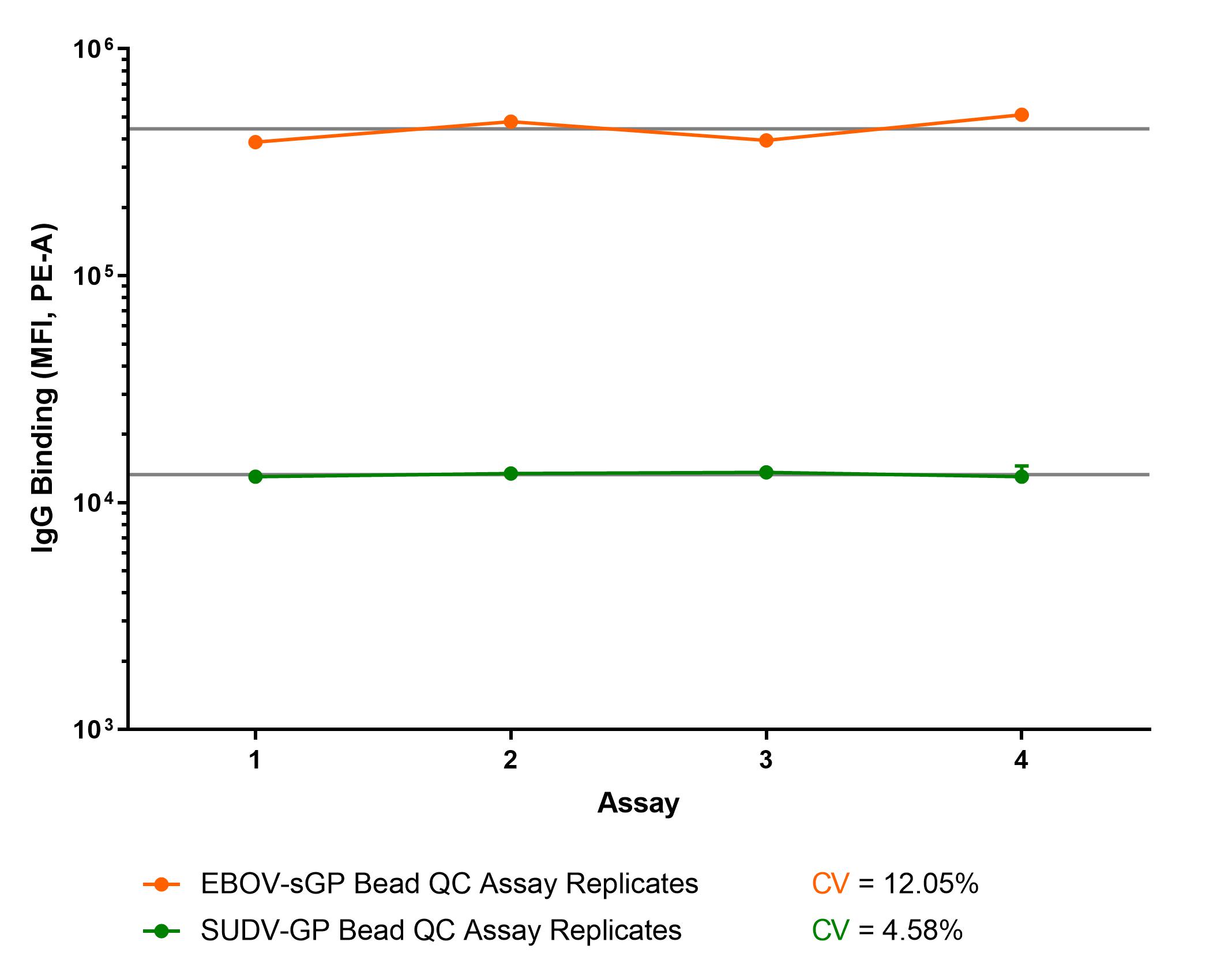 |
| --- | --- |
| **(C)**  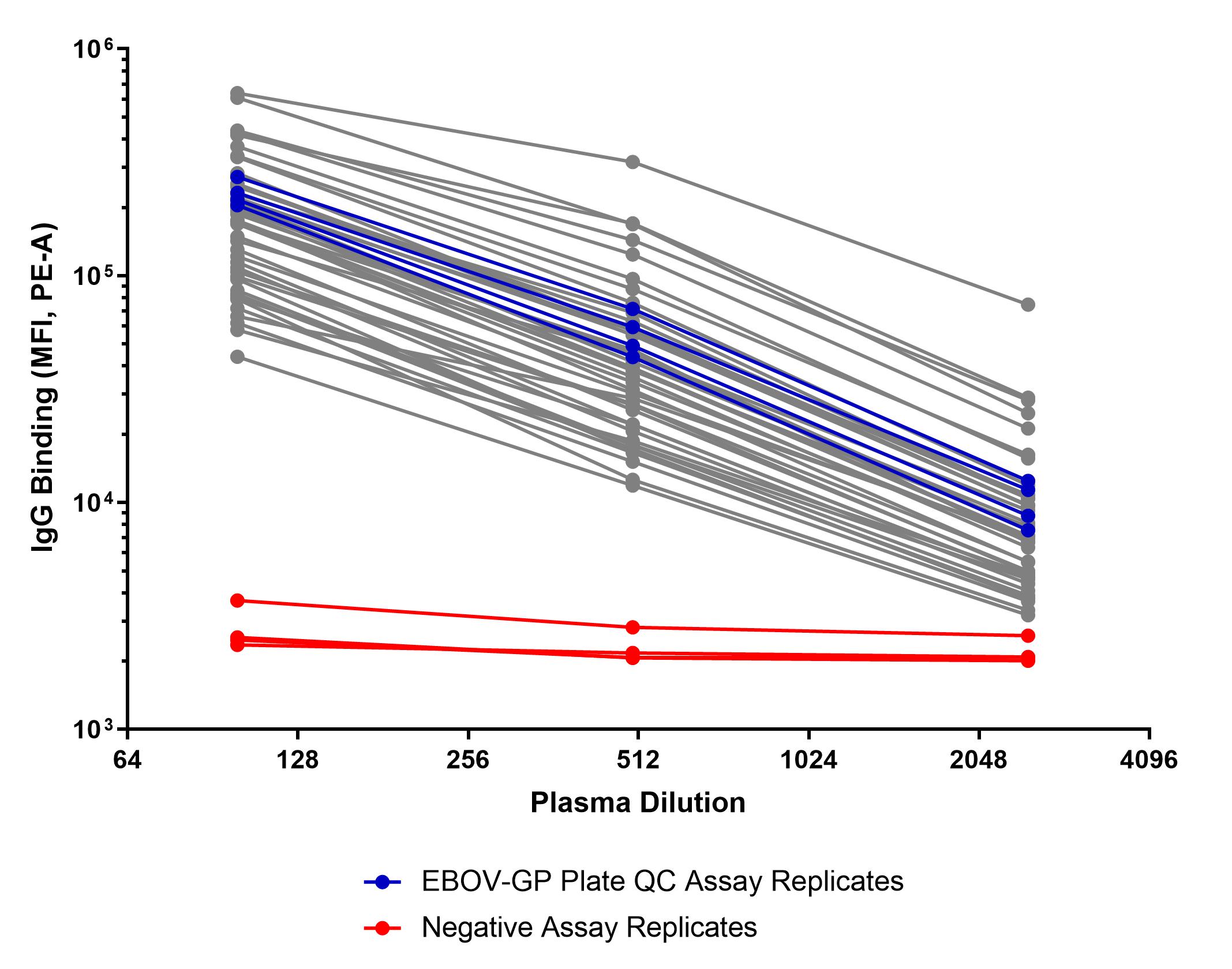 | **(D)**  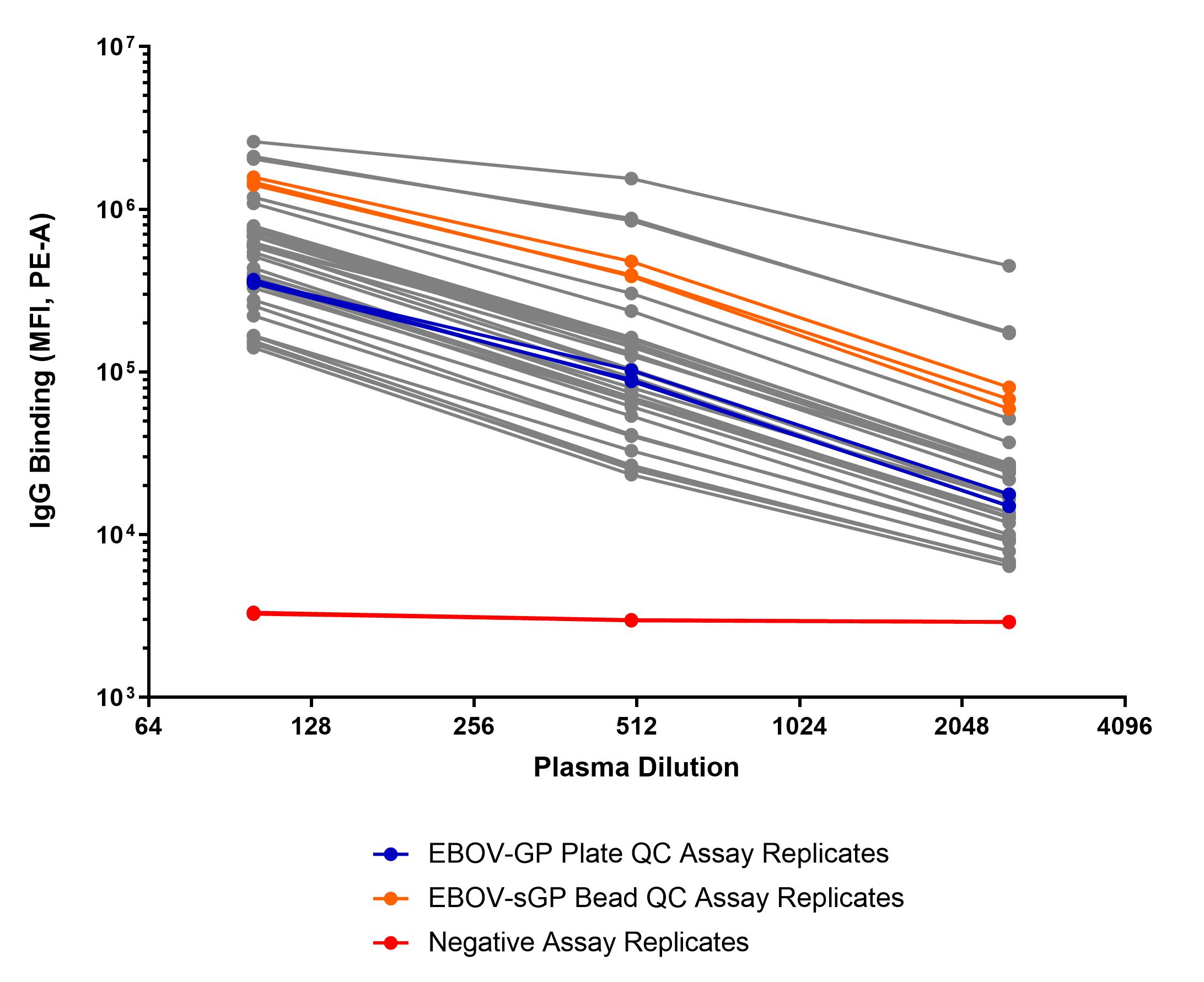 |
| **(E)**  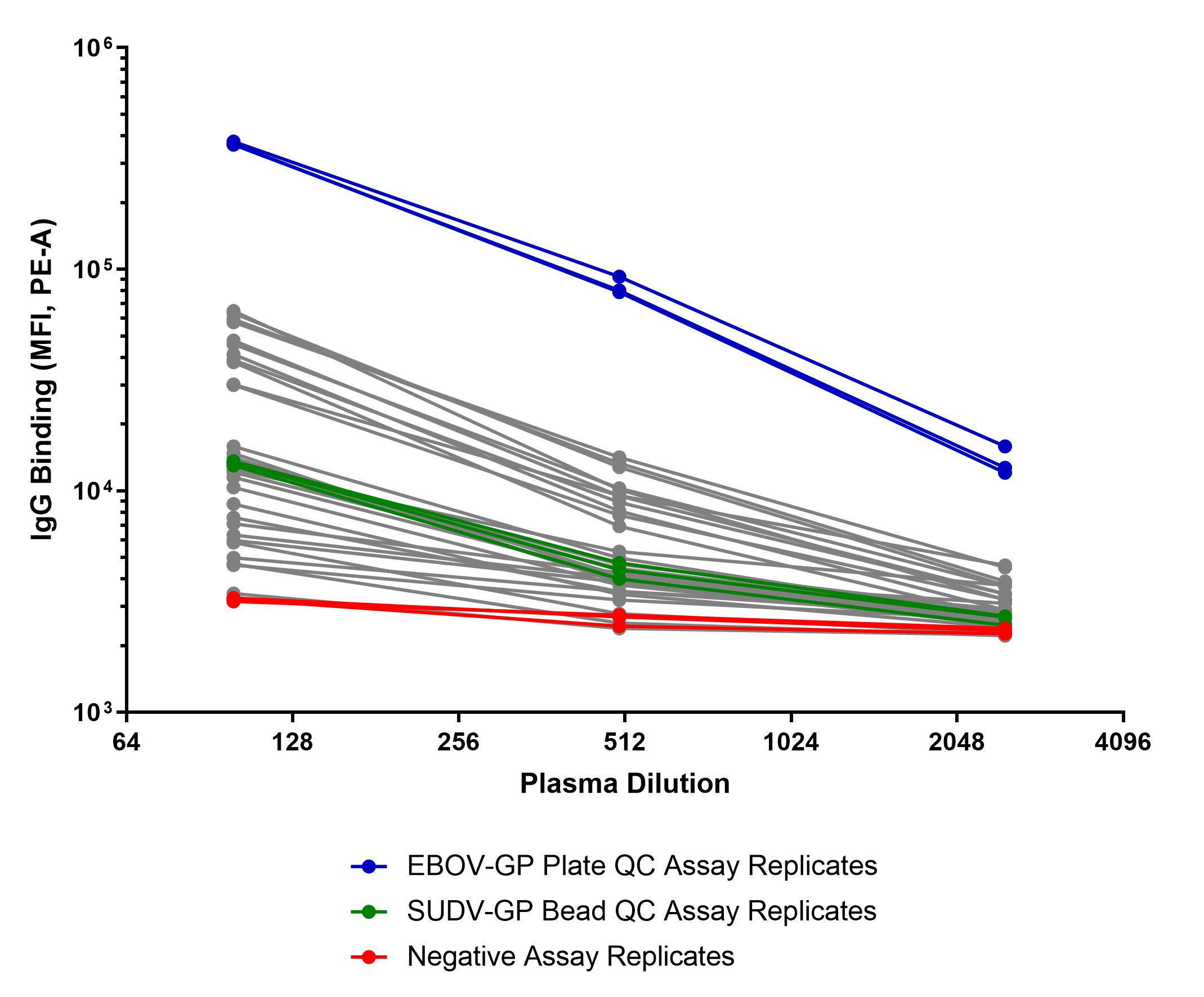 |  |

**Supplementary Figure 3: Raw MFI data and quality controls for flow cytometry IgG binding assays**

**(A)** Each plate used to detect IgG binding included a quality control sample using EBOV-GP beads with convalescent plasma at 1:100, 1:500, and 1:2500 dilutions, with a CV < 30%. **(B)** Further controls were included in the SUDV-GP and EBOV-sGP assays to monitor bead integrity. Raw MFI values for the IgG binding of all samples against **(C)** EBOV-GP, **(D)** EBOV-sGP, and **(E)** SUDV-GP were determined using FlowJO software (Version 10.8.0) and presented using GraphPad software (Version 9). The plasma samples tested are represented as grey dots.

| **(A)** 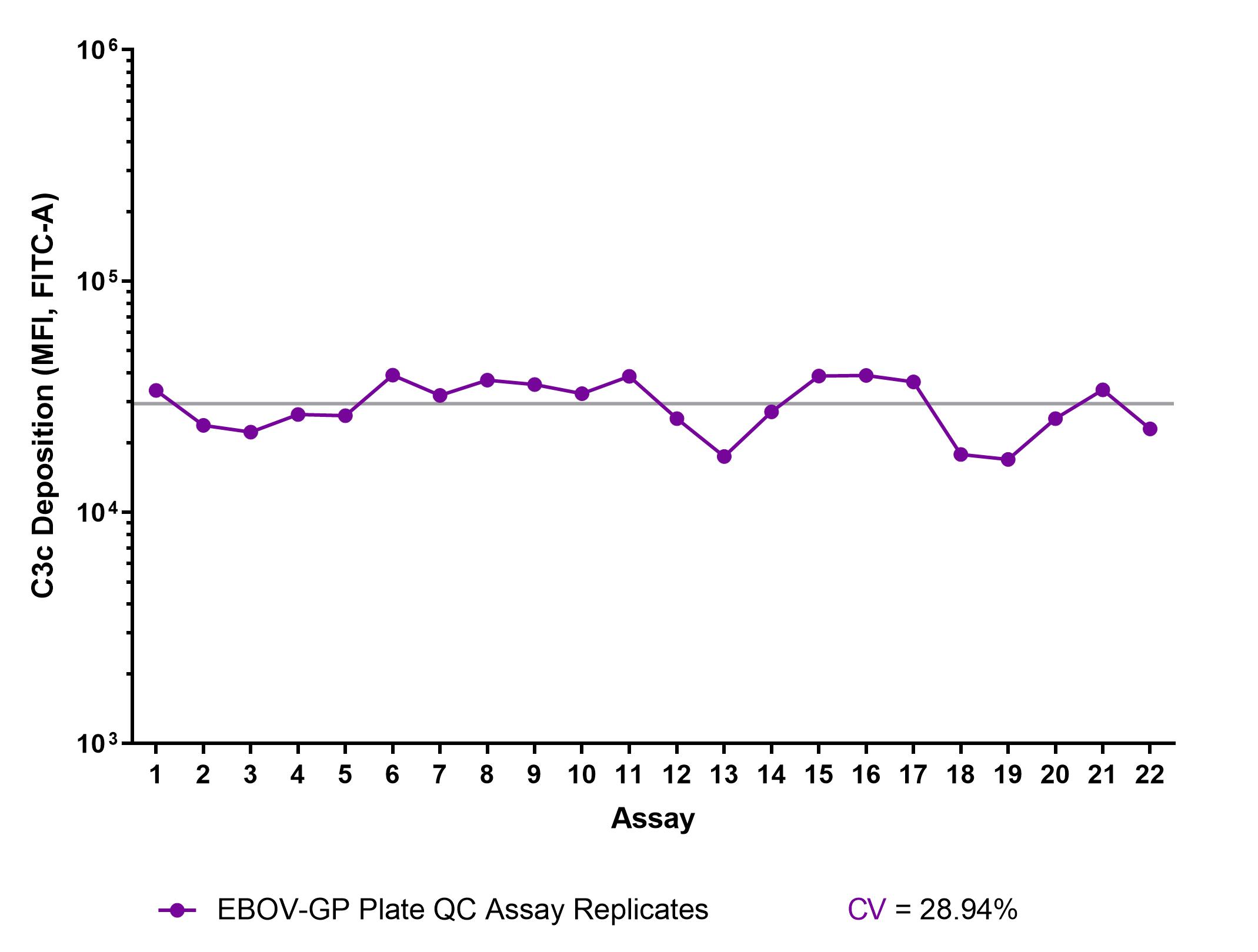 | **(B)**  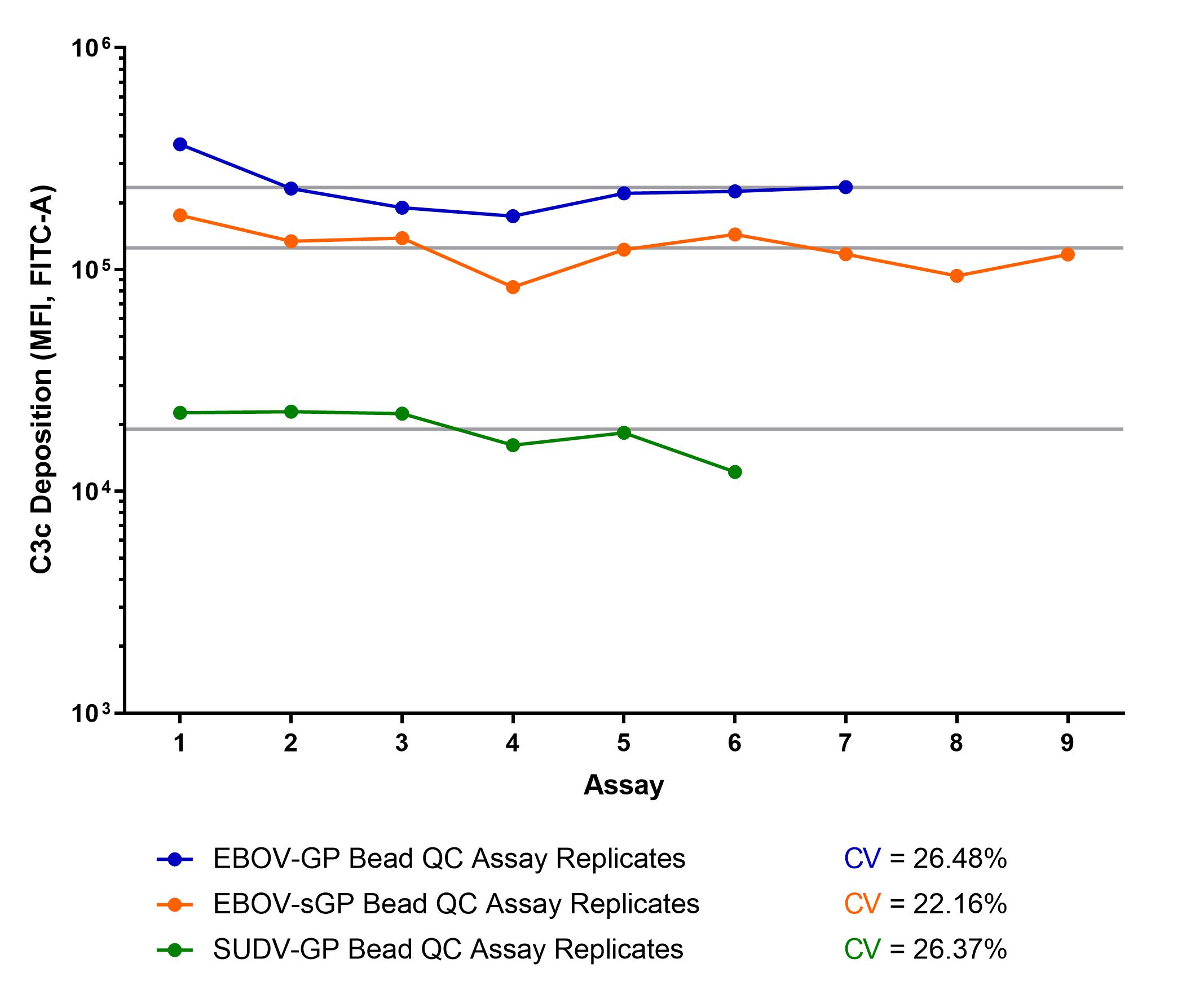 |
| --- | --- |
| **(C)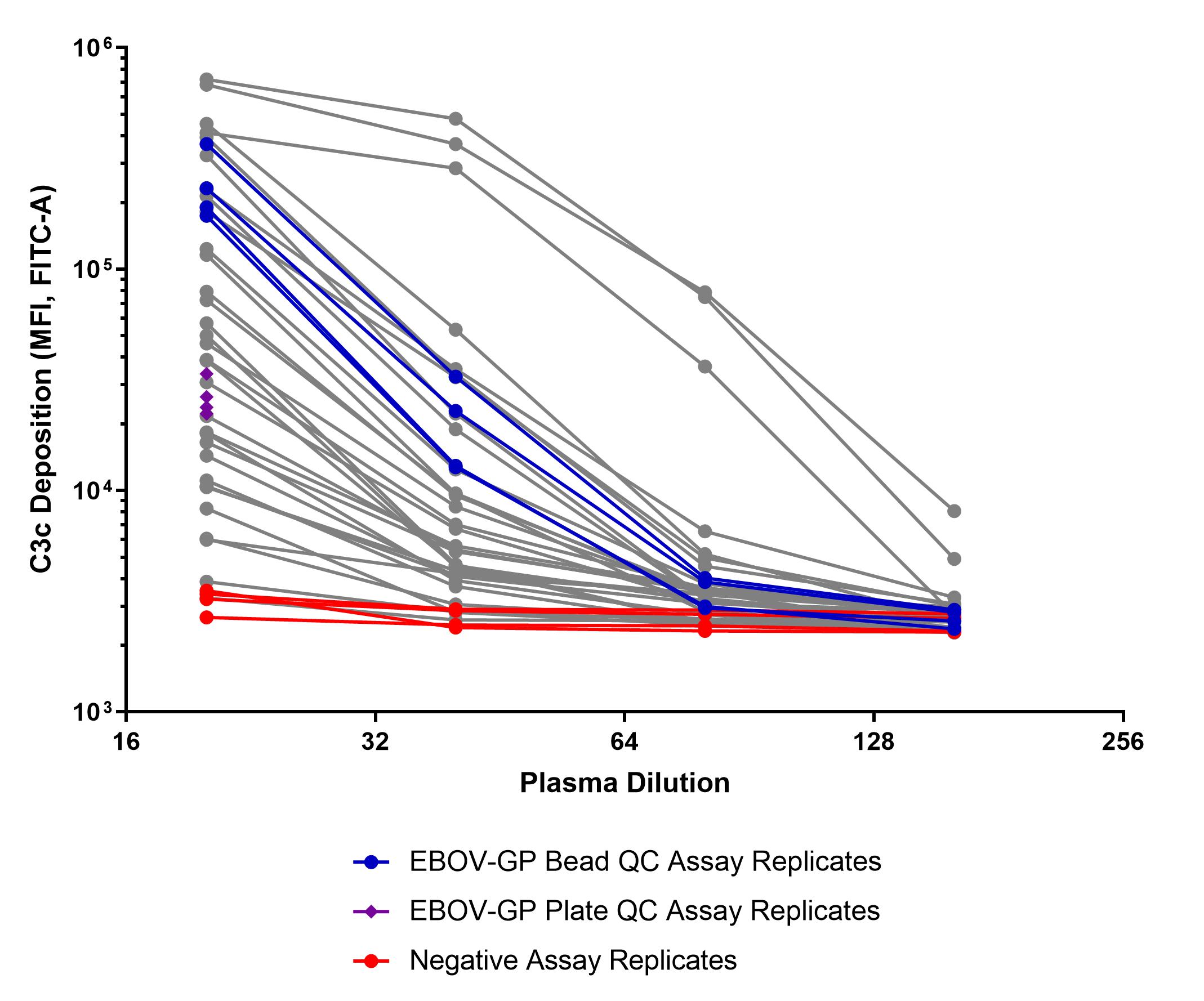** | **(D)**  **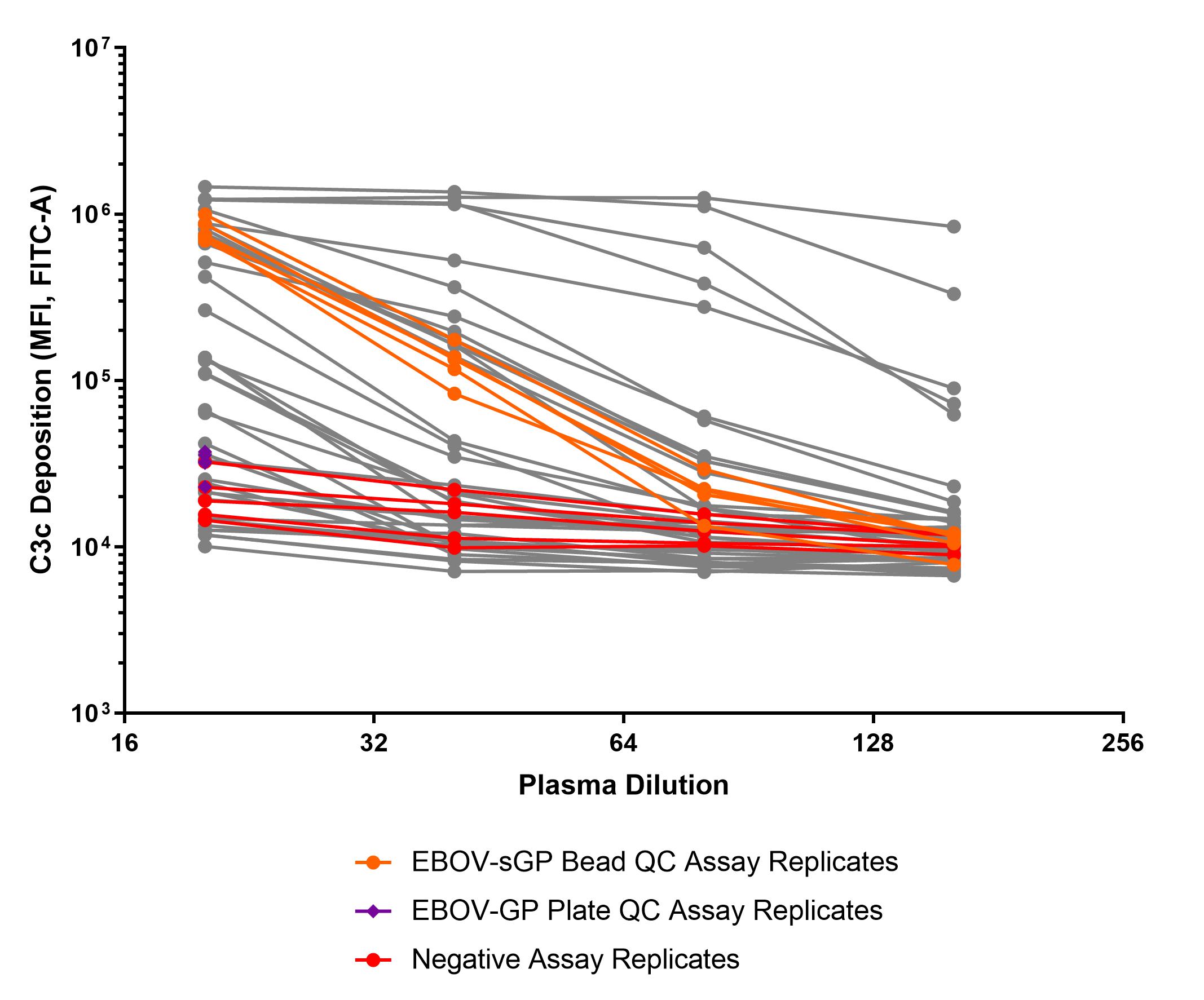** |
| **(E)**  **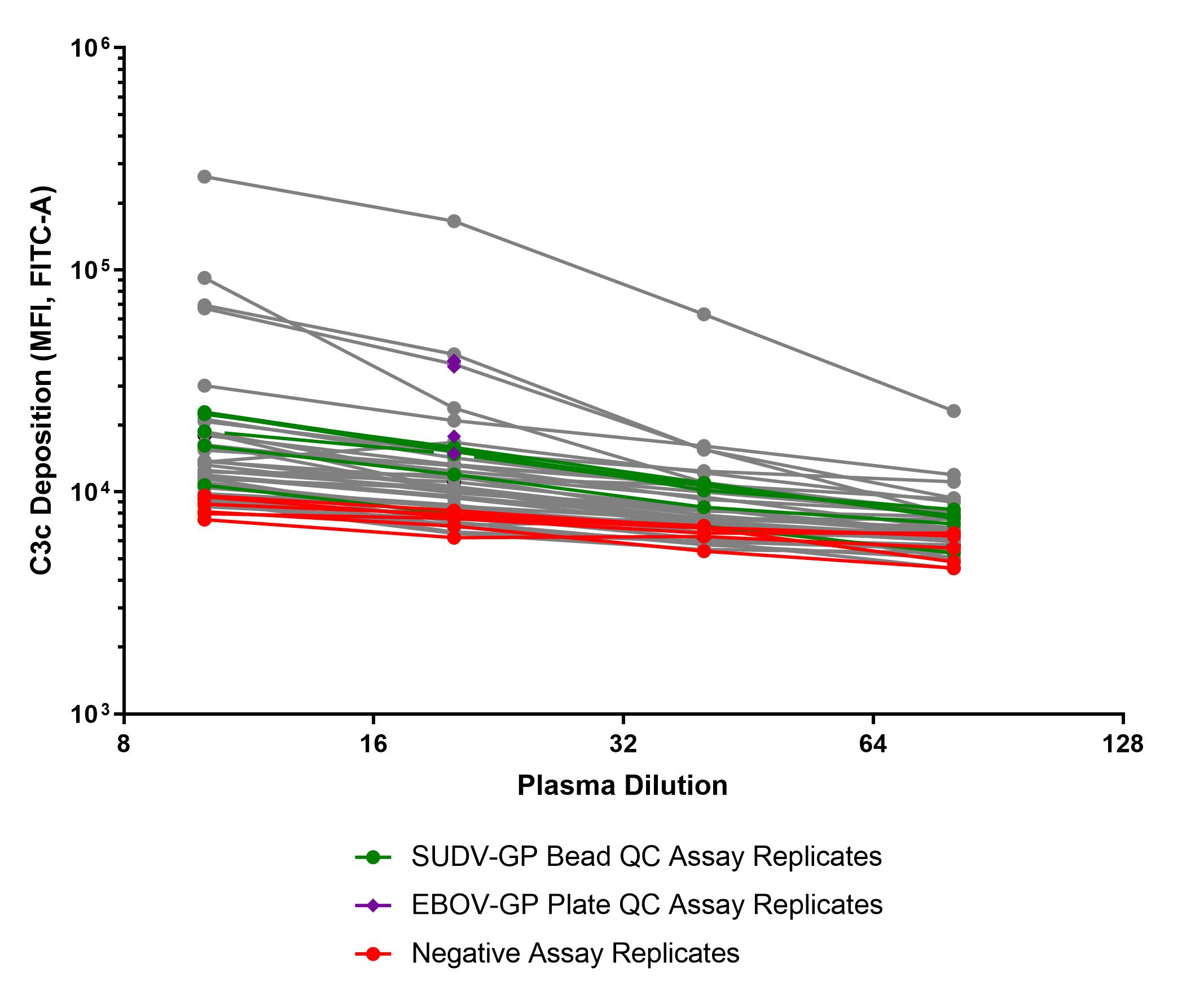** |  |

**Supplementary Figure 4:** **Raw MFI data and quality controls for flow cytometry C3c deposition assays**

**(A)** Each plate used to detect C3c deposition included a quality control sample using EBOV-GP beads with convalescent plasma at a fixed 1:20 dilution, with a CV cut-off <30%. **(B)** Further controls were included to monitor integrity of the relevant bead conjugates using the MFI from a 1:10 (SUDV-GP) or 1:20 (EBOV-GP, EBOV-sGP) plasma dilution with a CV <30%. Raw MFI values for C3c deposition of all samples against **(C)** EBOV-GP, **(D)** EBOV-sGP, and **(E)** SUDV-GP were determined using FlowJO software (Version 10.8.0) and presented using GraphPad software (Version 9). The plasma samples tested are represented as grey dots.

| **(A)**  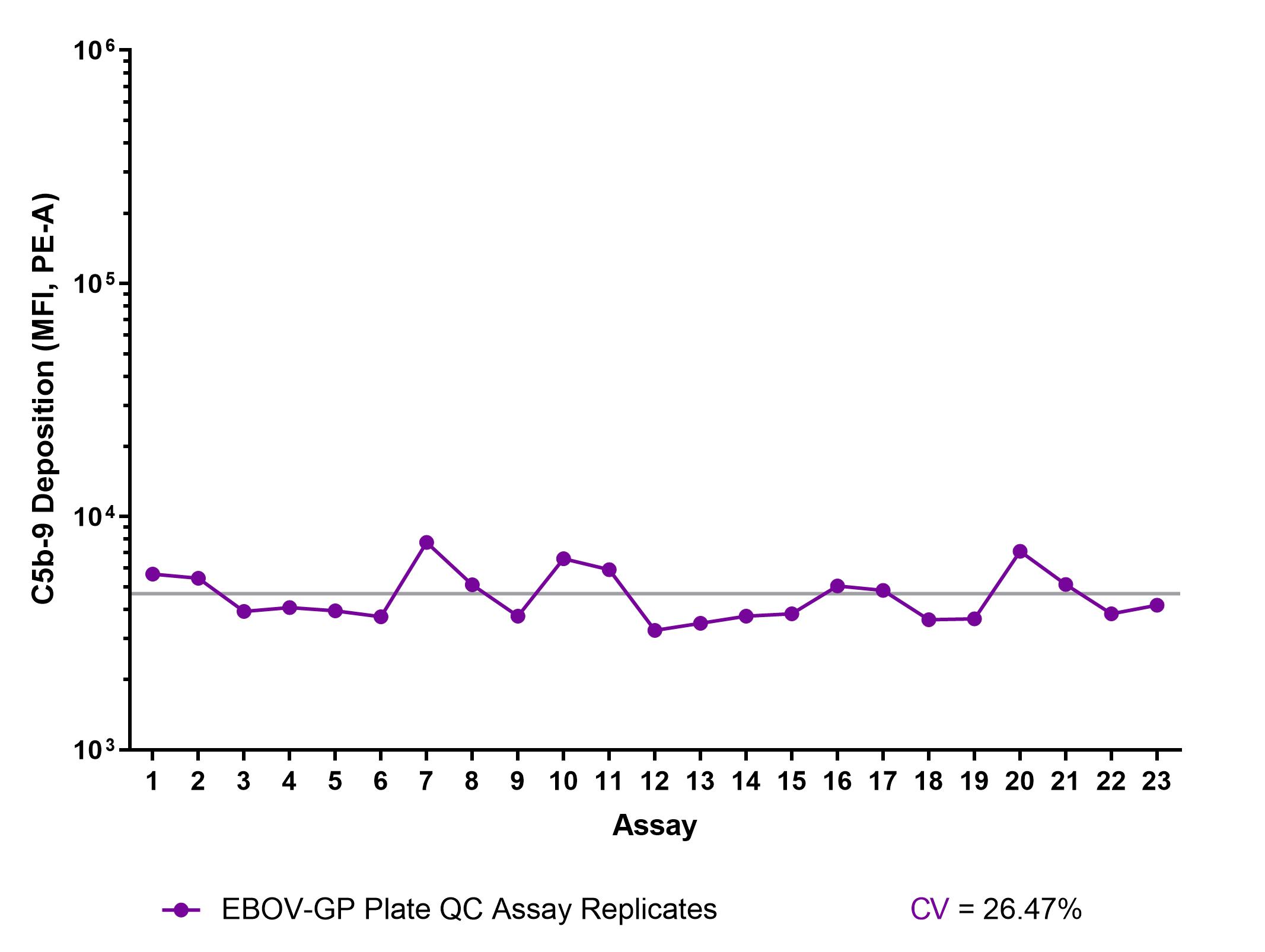 | **(B)**  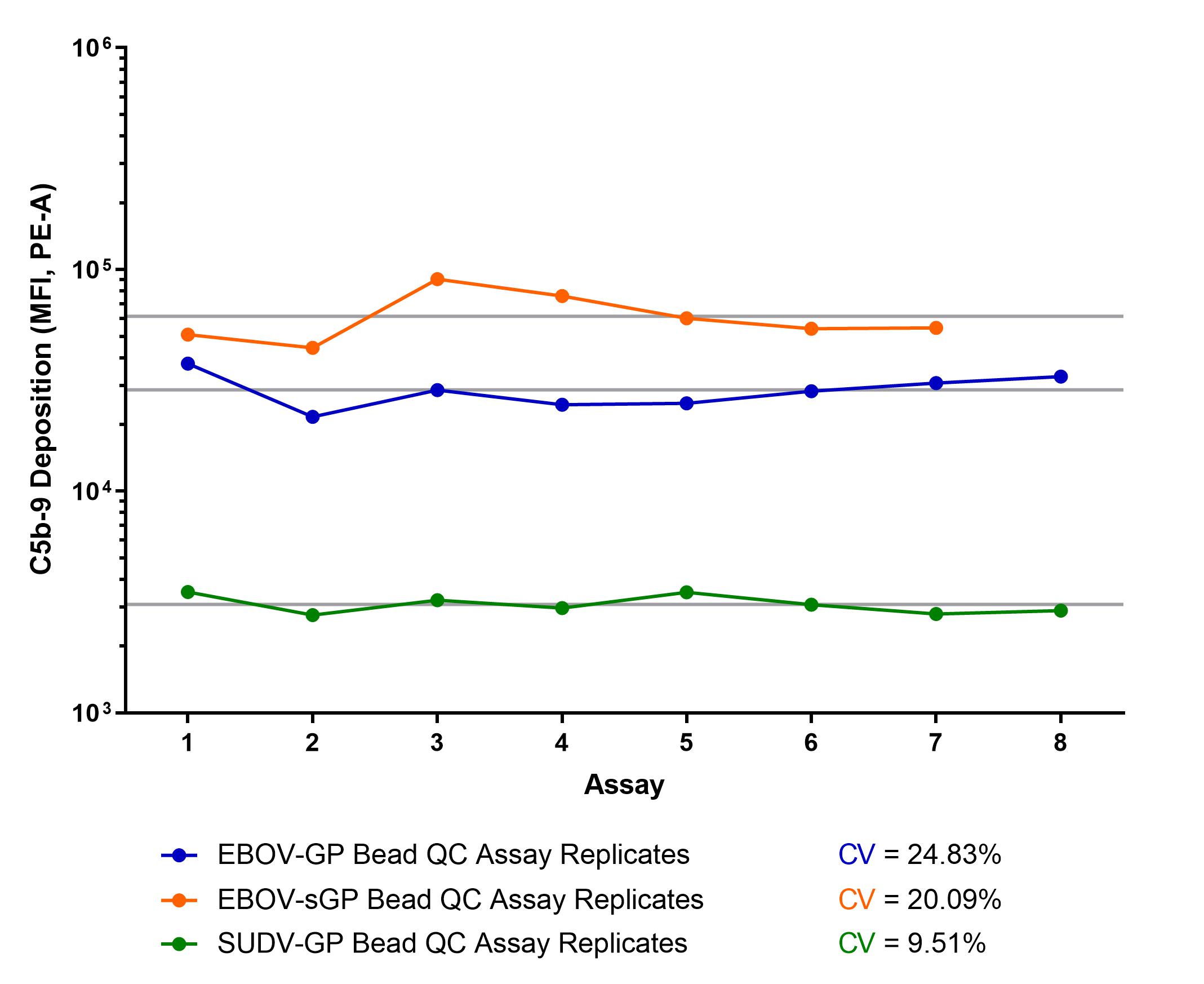 |
| --- | --- |
| **(C)**  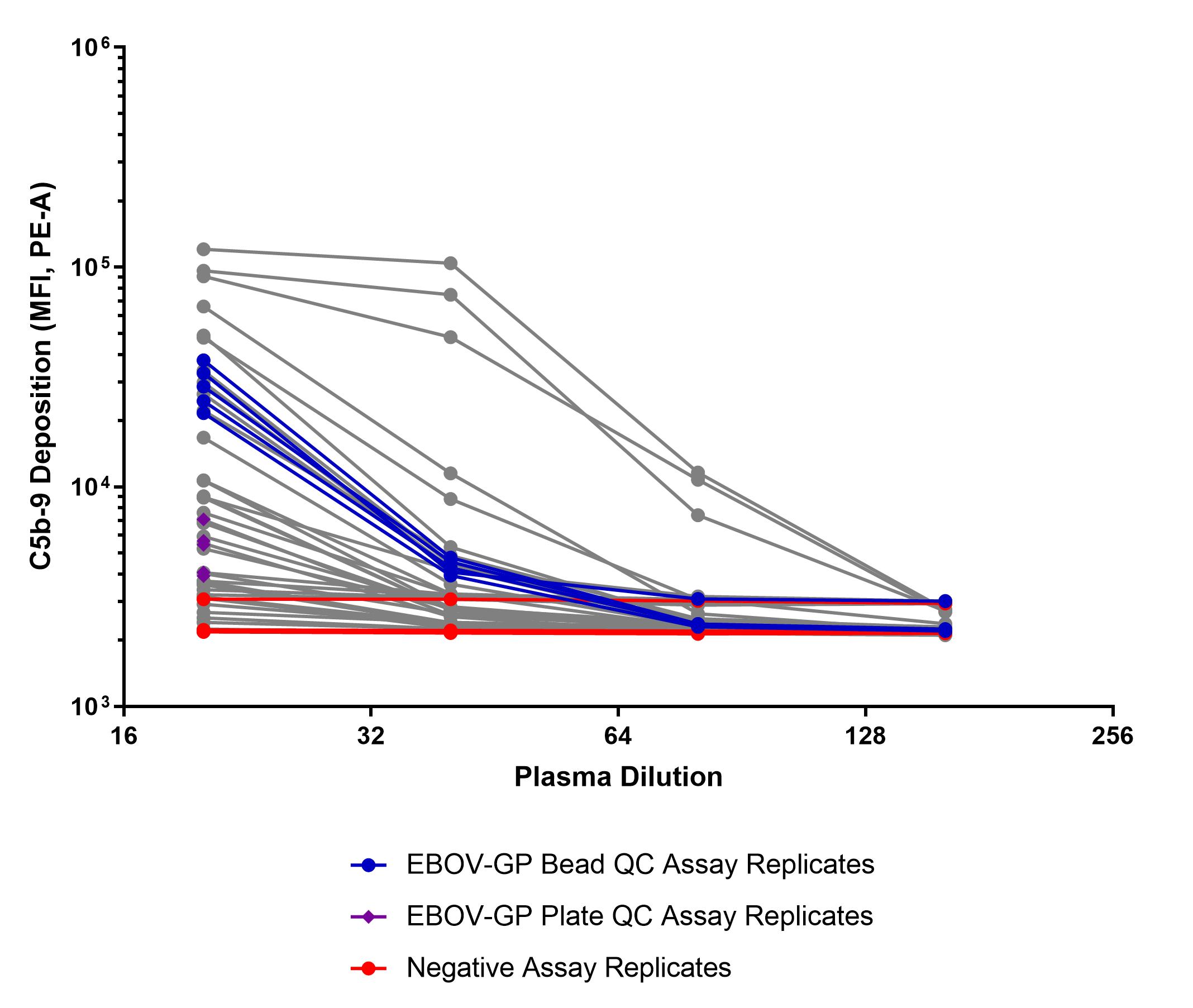 | **(D)**  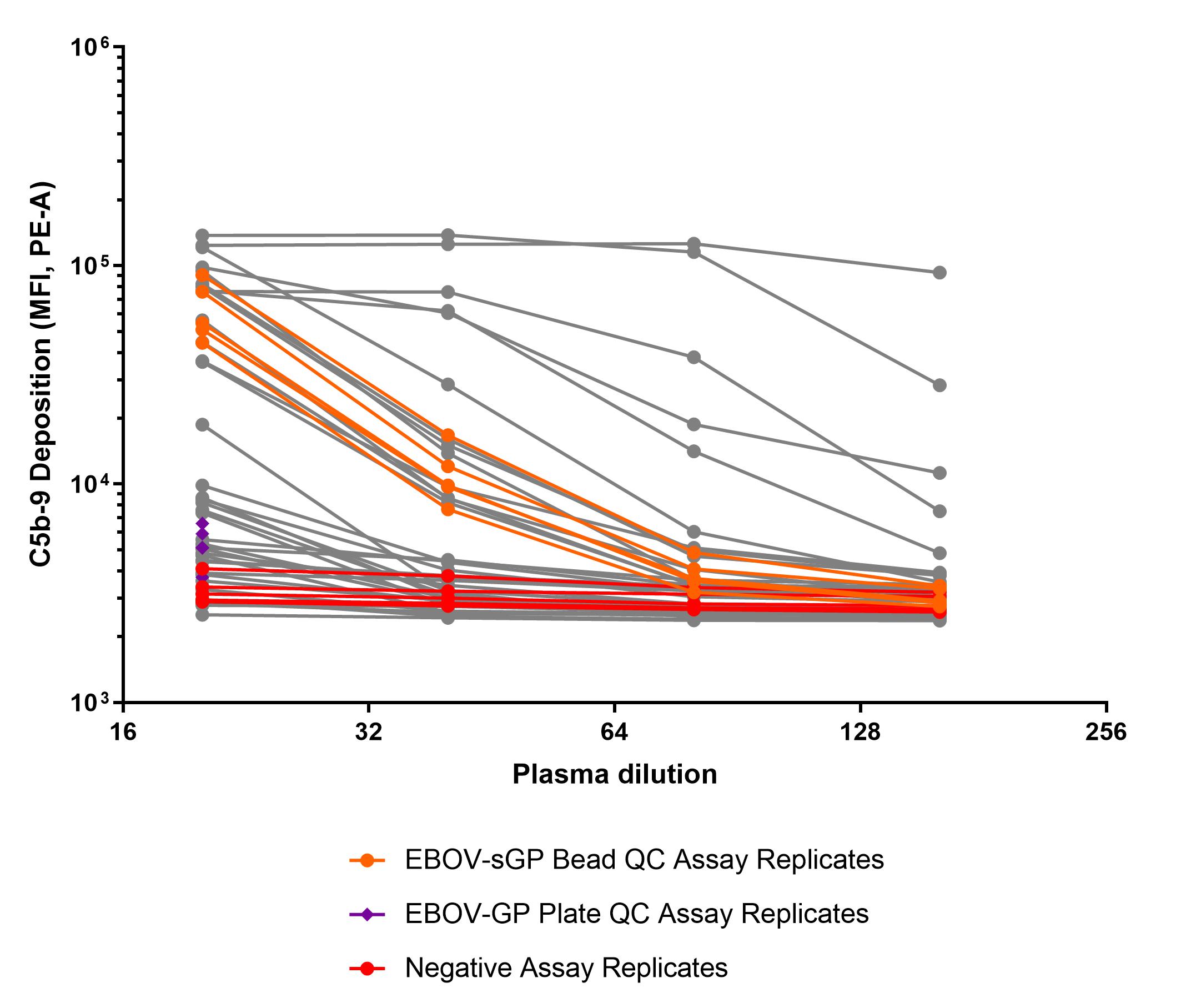 |
| **(E)**  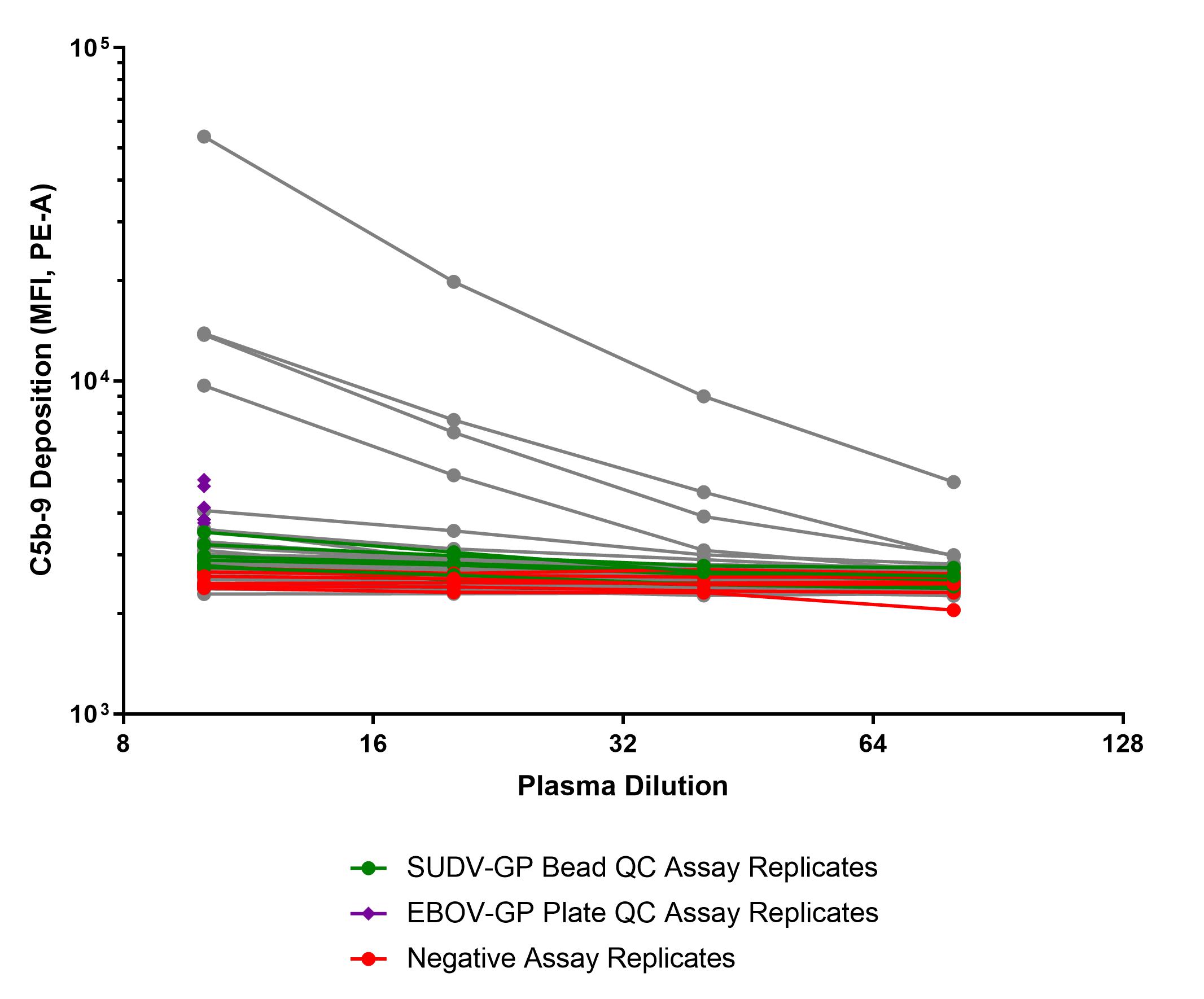 |  |

**Supplementary Figure 5:** **Raw MFI data and quality controls for flow cytometry C5b-9 deposition assays**

**(A)** Each plate used to detect C5b-9 deposition included a quality control sample using EBOV-GP beads with convalescent plasma at a fixed 1:20 dilution, with a CV cut-off <30%. **(B)** Further controls were included to monitor integrity of the relevant bead conjugates using the MFI from a 1:10 (SUDV-GP) or 1:20 (EBOV-GP, EBOV-sGP) plasma dilution with a CV <30%. Raw MFI values for C5b-9 deposition for all samples against **(C)** EBOV-GP, **(D)** EBOV-sGP, and **(E)** SUDV-GP were determined using FlowJO software (Version 10.8.0) and presented using GraphPad software (Version 9). The plasma samples tested are represented as grey dots.

| 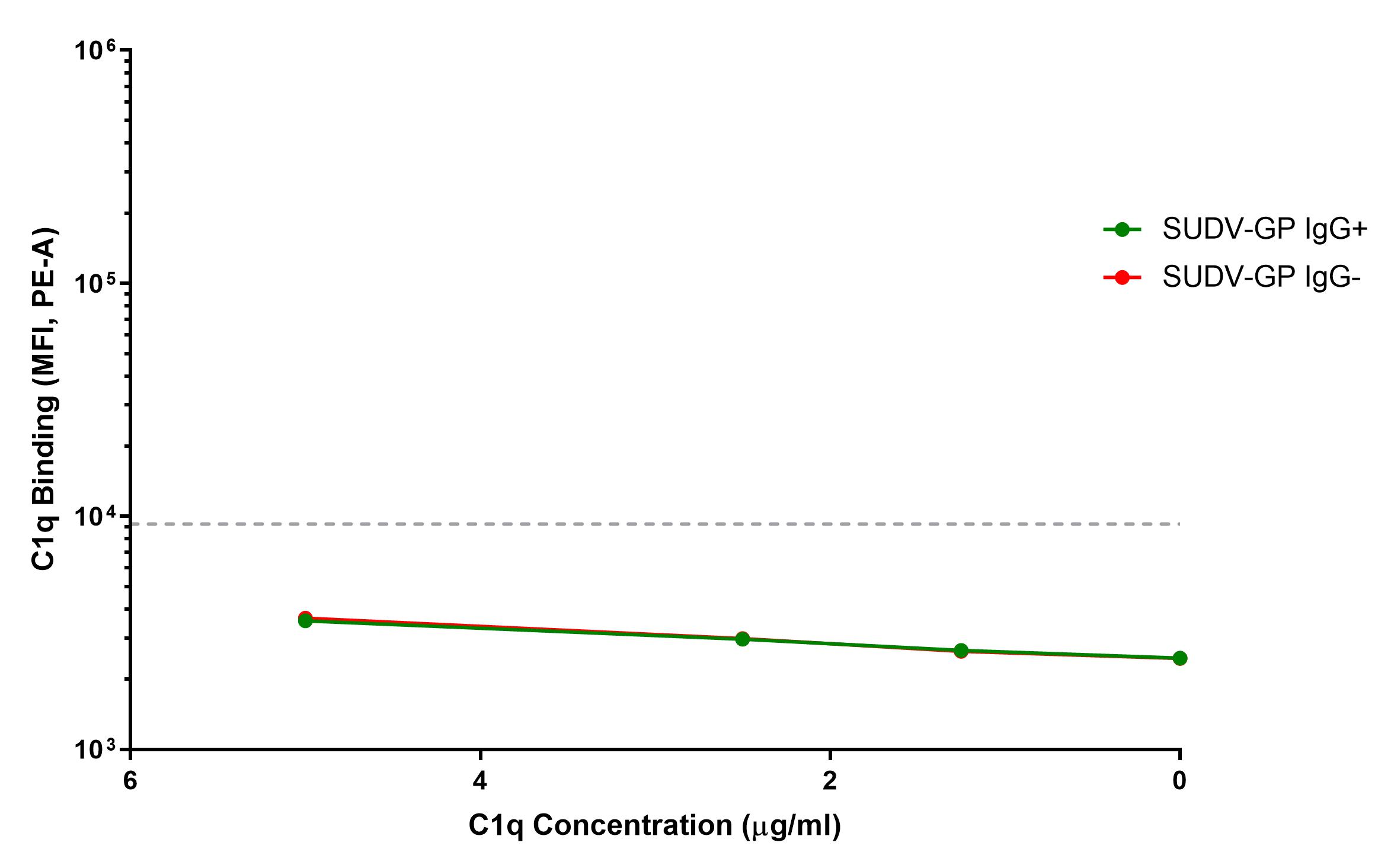 |
| --- |

**Supplementary Figure 6**: **The binding of purified C1q protein to SUDV-GP in antibody complexes**

Purified C1q protein was titrated against SUDV-GP conjugated beads with human plasma in the presence or absence of EBOV-GP IgG. A negative cut-off (grey dotted line) was determined using the mean value for all control samples where the primary antibody was excluded, plus three standard deviations. The assay signal did not exceed the cut-off in any of the conditions tested.

| **Cell Control** | Without PHP   | 10% PHP   | 20% PHP   | 40% PHP   |
| --- | --- | --- | --- | --- |

**Supplementary Figure 7: Microscopy of Vero cells used in wild-type EBOV neutralisation assays supplemented with pooled human plasma**

Vero cells were imaged to monitor the potential cytopathic effects (CPE) from the addition of pooled human plasma (PHP). Visible morphological differences in the cells became evident when the concentration of PHP was increased to 40%.
